# *Bacillus anthracis’* lethal toxin induces broad transcriptional responses in human peripheral monocytes

**DOI:** 10.1186/1471-2172-13-33

**Published:** 2012-07-02

**Authors:** Kassidy M Chauncey, M Cecilia Lopez, Gurjit Sidhu, Sarah E Szarowicz, Henry V Baker, Conrad Quinn, Frederick S Southwick

**Affiliations:** 1 Department of Medicine, University of Florida College of Medicine, Gainesville, FL, 32610, USA; 2 USA, Department of Molecular Genetics and Microbiology, University of Florida, Gainesville, FL, 32610, USA; 3 Centers for Disease Control and Prevention, Atlanta, GA, 30333, USA

## Abstract

**Background:**

Anthrax lethal toxin (LT), produced by the Gram-positive bacterium *Bacillus anthracis*, is a highly effective zinc dependent metalloprotease that cleaves the N-terminus of mitogen-activated protein kinase kinases (MAPKK or MEKs) and is known to play a role in impairing the host immune system during an inhalation anthrax infection. Here, we present the transcriptional responses of LT treated human monocytes in order to further elucidate the mechanisms of LT inhibition on the host immune system.

**Results:**

Western Blot analysis demonstrated cleavage of endogenous MEK1 and MEK3 when human monocytes were treated with 500 ng/mL LT for four hours, proving their susceptibility to anthrax lethal toxin. Furthermore, staining with annexin V and propidium iodide revealed that LT treatment did not induce human peripheral monocyte apoptosis or necrosis. Using Affymetrix Human Genome U133 Plus 2.0 Arrays, we identified over 820 probe sets differentially regulated after LT treatment at the p <0.001 significance level, interrupting the normal transduction of over 60 known pathways. As expected, the MAPKK signaling pathway was most drastically affected by LT, but numerous genes outside the well-recognized pathways were also influenced by LT including the IL-18 signaling pathway, Toll-like receptor pathway and the IFN alpha signaling pathway. Multiple genes involved in actin regulation, signal transduction, transcriptional regulation and cytokine signaling were identified after treatment with anthrax LT.

**Conclusion:**

We conclude LT directly targets human peripheral monocytes and causes multiple aberrant gene responses that would be expected to be associated with defects in human monocyte’s normal signaling transduction pathways and function. This study provides further insights into the mechanisms associated with the host immune system collapse during an anthrax infection, and suggests that anthrax LT may have additional downstream targets outside the well-known MAPK pathway.

## Background

*Bacillus anthracis*, the causative agent of anthrax, is a gram-positive bacterium that is naturally found in the soil, and rarely affects the human population. Unfortunately, deliberate dissemination of anthrax spores is capable of delivering a highly potent and lethal air-borne bioterrorist agent, as documented in the 2001 U.S. anthrax attacks. Inhalation anthrax is a highly fatal, acute disease characterized by a rapid onset of systemic shock and ultimately death [1].

The most virulent strains of *B. anthracis* contain two plasmids, pXO2 and pXO1, encoding an antiphagocytic poly-D-glutamic acid capsule and three exotoxins: lethal factor, edema factor and protective antigen [2]. Protective antigen is an 83 kDa protein that is known to bind to two host cell receptors, TEM-8 and CMG-2, facilitating the entry of edema and/or lethal factor into host cells [3]. Lethal factor is a 90 kDa zinc-dependent metalloprotease that cleaves the N-terminus of mitogen-activated protein kinase kinases (MAPKKs or MEKs) [4,5]. Edema factor is an 89 kDa adenylate cyclase that increases intracellular cAMP levels [6].

Previous studies using anthrax animal models have documented resistance to anthrax lethal toxin (LT) through depletion of host macrophages, suggesting that these cells play a critical role in anthrax LT induced lethality [7,8]. LT has also been shown to suppress cytokine responses by peripheral blood mononuclear cells, induce macrophage apoptosis, and prevent monocyte proliferation and differentiation [1,9,10]. Inhalation anthrax cases present clinical manifestations indicative of host immune collapse in humans and in nonhuman primate studies [11-13]. However, more recent studies investigating human monocytes and macrophages have suggested human alveolar macrophages are resistant to LT, and undifferentiated human monocytic cell lines are resistant to LT-induced death [10,14]. LT’s targeting of human monocytes/macrophages could help to explain the rapid onset of fatal symptoms and host demise during an inhalation anthrax infection, but the exact effects LT exerts on human peripheral monocytes, along with the mechanisms underlying the impairment of the host immune cell’s responses, have yet to be fully determined.

Previous studies investigating LT treated murine macrophages have shown a broad range in transcriptional effects induced by LT. These studies concluded LT-induced changes in macrophage inflammation, signaling, and transcription factors, along with changes in the immune response by macrophages. This study discovered the down regulation of CD-137 after LT treatment, shown to play a role in monocyte proliferation in response to LPS, and up regulation of plasminogen activator inhibitor type I, which results in fibrin deposits, massive imbalances in coagulation, and, in some instances, multi-organ failure [15,16]. Another study has measured the transcriptional responses of THP-1 cells after *B. anthracis* spore exposure, finding toxigenic *B. anthracis* strains suppress the cell signaling responses to infection [17].

Blood monocytes are mononuclear cells that play a major role in the host immune response through regulation of inflammatory responses, secretion of cytokine and antimicrobial factors, and direct pathogen clearance [18]. Monocytes are derived from monoblasts in the bone marrow, and circulate in the blood for 1-2 days before they migrate into tissues where they replenish the macrophage and dendritic pools [19-21]. Here, we determined human monocyte susceptibility to LT by demonstrating cleavage of MEKs, and utilized Affymetrix GeneChip® Human Genome U133 Plus 2.0 Arrays in order to identify additional mechanisms of LT impairment on the transcriptional responses of human peripheral monocytes. The arrays contained 54,675 probe sets representing over 22,000 of the best characterized human genes, providing extensive insights into the mechanisms behind LT induced dysfunction of human peripheral monocytes.

This study is the first to determine direct human monocyte susceptibility via cleavage of MEKs, along with the analysis of the transcriptional responses, to anthrax LT. The mechanisms of LT impairment on human peripheral monocytes will help elucidate the roles monocytes contribute during the host immune system collapse documented during an anthrax infection. The transcriptional analysis will serve to not only unravel the mechanisms behind the rapid onset of death in anthrax victims, but will also potentially provide new targets for controlling inflammation and enhancing host defense.

## Results and discussion

### Monocyte purity, apoptosis and susceptibility to anthrax LT

**Figure 1:**
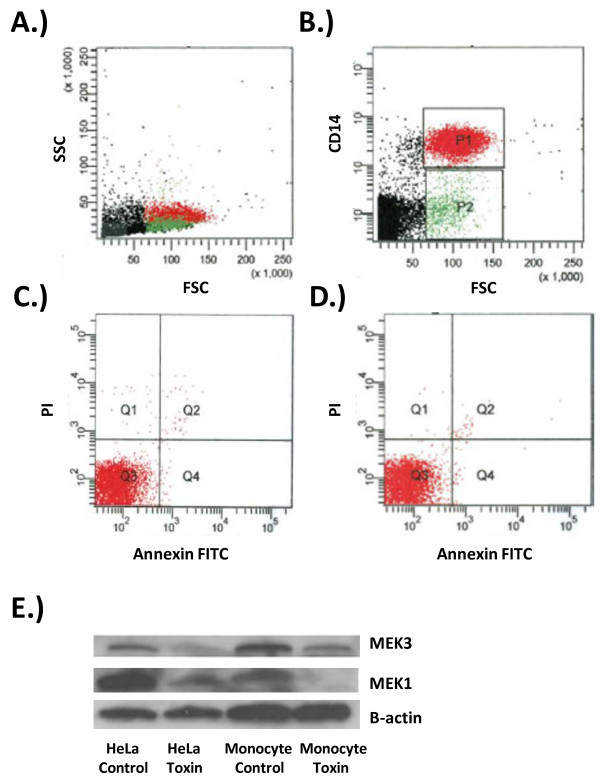
**Monocyte purity, apoptosis, and susceptibility to LT. **Red = CD14+ monocytes. Green = CD14-lymphocytes. **A.)** Forward and side scatter analysis of purified fixed human monocytes showing the monocyte population as compared to total population. **B.)** CD14 Pacific Blue and forward scatter analysis of fixed purified human monocytes showing >85% monocytes. **C.)** PI and annexin-FITC analysis of CD14 + monocytes after a 4 h incubation showing 99.0% viable cells indicated in quadrant 3. **D.)** PI and annexin-FITC analysis of CD14 + monocytes after a 4 h LT treatment showing 99.1% viable cells indicated in quadrant 3. HeLa cells or human monocytes were left untreated or treated with 500 ng/mL LT for 4 h at 37°C. Samples were lysed, run on SDS-PAGE, transferred to PVDF membrane, and probed with indicated antibodies. Both MEK3 and MEK1 were cleaved by LT while control cells showed no MEK cleavage. β-actin loading controls show equivalent loading of both control and LT treated cells.

In order to first determine monocyte cell purity, isolated cells were analyzed using flow cytometry and gated using forward and side scatter, along with the monocytic marker, CD14. It was found that monocytes were isolated with a >85% purity (Figure 1A and 1B). Because previous reports have documented LT induced cell apoptosis, it was important to assure the transcriptional response of LT treated monocytes were independent of apoptosis. This was assured by the analysis of the necrosis and apoptosis markers, propidium iodide (PI) and annexin V, on human peripheral monocytes. Nearly all (99%) human peripheral monocytes showed no evidence of necrosis or apoptosis after a 4 h treatment of LT (Figure 1C and 1D). There has been some conflicting data suggesting monocytes, along with monocyte-derived cells, are not susceptible to the actions of anthrax LT. One study utilized human monocytic cell lines and found that undifferentiated monocytic cells did not undergo LT-induced cytotoxicity, while the differentiated cells were susceptible [10]. Another study investigating human alveolar macrophages (AM) found that these cells were relatively resistant to the actions of LT. It was ascertained that LT failed to suppress human AM cytokine responses, cleave MEKs, and induce apoptosis [14].

In order to explore the actions of LT on human peripheral monocytes, a Western Blot analysis was performed and MEK1, along with MEK3, cleavage was determined after a 4-hour treatment with LT. Human peripheral monocytes were found to be susceptible to the actions of LT as evidenced by cleavage of MEK1 and MEK3 (Figure 1E). HeLa cells were used as a positive control and β-actin was used to assure equal loading controls. We conclude that human peripheral monocytes are a direct target of anthrax LT.

### Microarray analysis and results

**Figure 2:**
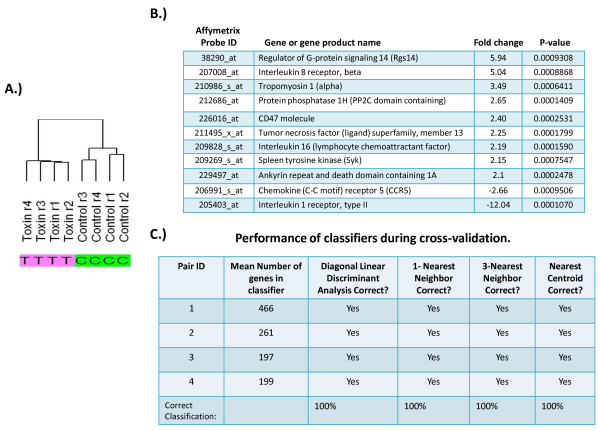
**Unsupervised microarray analysis. A.)** Hierarchical clustering dendrogram showing similarities between expression patterns within each condition. Specimens were paired based on donor, using 4 separate donors as indicated in replica r1 through r4. **B.)** Significant genes (p < 0.001) up or down regulated after LT treatment, along with their fold change, p-value and probe ID. **C.)** Leave-one-out-cross validation was used to calculate mis-classification rate that yielded a 100% correct classification between pairs.

Human peripheral monocytes were treated with LT or media alone, and microarray analysis was performed using four biological replicates from healthy volunteers. A total of 8 microarray hybridizations were employed and analyzed on Affymetrix Gene Chips®(HG U133 plus 2.0). The chips contained 54,675 probe sets and identified multiple differentially regulated pathways and genes by human peripheral monocytes after LT treatment. Unsupervised hierarchical analysis was used to assess the noise in the array experiments. First, probe sets whose signal intensity varied most in the data set were selected by applying a variation filter. Probes sets that displayed a coefficient of variation of greater than 0.5 were subjected to hierarchical analysis. The clustering dendrogram showed the major node of separation between control and LT treated samples (Figure 2A).

**Figure 3:**
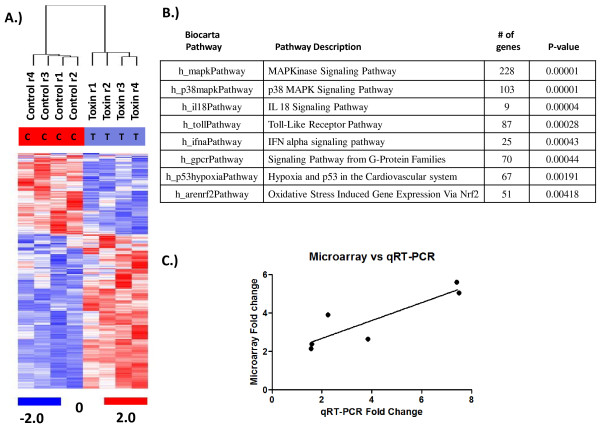
**Supervised microarray analysis. A.)** Hierarchical cluster analysis showing the 820 probe sets which were differentially expressed at the 0.001 significance level. The arrays clustering on the left are from control samples, whereas the cluster on the right shows the LT treated samples. Up-regulated genes are shown in red and down-regulated genes are shown in blue. **B.)** Biocarta pathway analysis showing the pathways most significantly affected by LT, along with the number of genes and p-value within each pathway that were affected. **C.)** Correlation of genes altered after treatment with anthrax LT using microarray analysis versus RT-PCR. Spearman correlation coefficient = 0.885.

To identify specific genes responsive to LT treatment, a paired *t*-test (by donor) was performed at a significance threshold of p < 0.001. Genes specified by 820 probe sets were found to be significant among the treatment groups (Table 1). The hierarchical cluster pattern of the significant probe sets is shown (Figure 3A). Of these probe sets, multiple gene products known to play a role in monocyte function were discovered (Figure 2B). The ability of probe sets significant at p < 0.001 to function as a classifier between treatment groups (LT treated vs. control) was established by leave-one-out-cross-validation and Monte Carlo simulations. Using 4 different prediction models, the classifier performed flawlessly. Of the significant genes identified, many are known to play a role in monocyte function (Figure 2C).

**Table 1:** Control vs Toxin corresponding P-value p<0.001

	**Paramet. p-value**	**Geometric mean of intensities**	**Probe set**	**Gene symbol**	**Description**
1	2.80E-006	1.78	222001_x_at	FAM91A2	family with sequence similarity 91, member A2
2	2.90E-006	1.25	218734_at	NAT11	N-acetyltransferase 11
3	3.40E-006	1.6	230350_at	NA	NA
4	7.80E-006	1.56	228930_at	NA	NA
5	8.70E-006	1.58	208661_s_at	TTC3	tetratricopeptide repeat domain 3
6	9.80E-006	1.16	218716_x_at	MTO1	mitochondrial translation optimization 1 homolog (S. cerevisiae)
7	1.07E-005	1.15	238538_at	ANKRD11	ankyrin repeat domain 11
8	1.22E-005	2.92	225896_at	NA	NA
9	1.41E-005	3.87	227450_at	ERP27	endoplasmic reticulum protein 27 kDa
10	1.49E-005	1.23	226602_s_at	BCR	breakpoint cluster region
11	1.60E-005	1.65	209123_at	QDPR	quinoid dihydropteridine reductase
12	1.66E-005	1.73	213934_s_at	ZNF23	zinc finger protein 23 (KOX 16)
13	1.66E-005	1.56	226419_s_at	SFRS1	splicing factor, arginine/serine-rich 1
14	1.84E-005	2.91	227946_at	OSBPL7	oxysterol binding protein-like 7
15	2.04E-005	1.84	242989_at	NA	NA
16	2.18E-005	1.92	242590_at	NA	NA
17	2.29E-005	1.36	204559_s_at	LSM7	LSM7 homolog, U6 small nuclear RNA associated (S. cerevisiae)
18	2.35E-005	1.68	225902_at	NA	NA
19	2.44E-005	1.29	220939_s_at	DPP8	dipeptidyl-peptidase 8
20	2.63E-005	1.2	218682_s_at	SLC4A1AP	solute carrier family 4 (anion exchanger), member 1, adaptor protein
21	2.72E-005	2.21	212056_at	KIAA0182	KIAA0182
22	2.91E-005	2.87	222477_s_at	TM7SF3	transmembrane 7 superfamily member 3
23	3.01E-005	2.01	202512_s_at	ATG5	ATG5 autophagy related 5 homolog (S. cerevisiae)
24	3.07E-005	1.52	209042_s_at	UBE2G2	ubiquitin-conjugating enzyme E2G 2 (UBC7 homolog, yeast)
25	3.10E-005	5.25	232181_at	LOC153346	hypothetical protein LOC153346
26	3.13E-005	1.79	1554452_a_at	HIG2	hypoxia-inducible protein 2
27	3.36E-005	2.11	228772_at	HNMT	histamine N-methyltransferase
28	3.37E-005	1.3	221501_x_at	LOC339047	hypothetical protein LOC339047
29	3.39E-005	1.81	239038_at	C1orf52	chromosome 1 open reading frame 52
30	3.46E-005	1.95	203839_s_at	TNK2	tyrosine kinase, non-receptor, 2
31	3.89E-005	1.89	227558_at	CBX4	chromobox homolog 4 (Pc class homolog, Drosophila)
32	3.90E-005	1.4	214691_x_at	FAM63B	family with sequence similarity 63, member B
33	3.91E-005	1.32	228301_x_at	NDUFB10	NADH dehydrogenase (ubiquinone) 1 beta subcomplex, 10, 22kDa
34	4.05E-005	1.8	1556306_at	NA	NA
35	4.22E-005	3.27	229016_s_at	TRERF1	transcriptional regulating factor 1
36	4.47E-005	3.85	223741_s_at	TTYH2	tweety homolog 2 (Drosophila)
37	4.48E-005	5.6	49306_at	RASSF4	Ras association (RalGDS/AF-6) domain family member 4
38	4.63E-005	1.49	32209_at	FAM89B	family with sequence similarity 89, member B
39	4.75E-005	2.83	225298_at	PNKD	paroxysmal nonkinesigenic dyskinesia
40	5.00E-005	1.59	228726_at	NA	NA
41	5.36E-005	1.17	1562984_at	NA	NA
42	5.39E-005	1.6	201639_s_at	CPSF1	cleavage and polyadenylation specific factor 1, 160kDa
43	5.66E-005	1.47	221649_s_at	PPAN	peter pan homolog (Drosophila)
44	5.88E-005	1.94	225360_at	TRABD	TraB domain containing
45	6.23E-005	1.27	221005_s_at	PTDSS2	phosphatidylserine synthase 2
46	6.35E-005	2.13	228914_at	NA	NA
47	6.47E-005	1.65	208206_s_at	RASGRP2	RAS guanyl releasing protein 2 (calcium and DAG-regulated)
48	6.49E-005	1.64	209198_s_at	SYT11	synaptotagmin XI
49	6.55E-005	1.7	221575_at	SCLY	selenocysteine lyase
50	6.74E-005	1.82	229969_at	NA	NA
51	6.76E-005	2.22	235513_at	NA	NA
52	6.77E-005	1.93	236922_at	NA	NA
53	6.78E-005	1.14	204364_s_at	REEP1	receptor accessory protein 1
54	6.87E-005	1.55	227025_at	PPHLN1	periphilin 1
55	7.02E-005	1.78	227288_at	SFRS12IP1	SFRS12-interacting protein 1
56	7.13E-005	2.55	205075_at	SERPINF2	serpin peptidase inhibitor, clade F (alpha-2 antiplasmin, pigment epithelium derived factor), member 2
57	7.16E-005	2.29	222988_s_at	TMEM9	transmembrane protein 9
58	7.35E-005	1.39	231831_at	COX19	COX19 cytochrome c oxidase assembly homolog (S. cerevisiae)
59	7.37E-005	1.88	221788_at	NA	NA
60	7.66E-005	1.61	236004_at	NA	NA
61	7.68E-005	1.85	219751_at	SETD6	SET domain containing 6
62	7.92E-005	1.74	227273_at	NA	NA
63	8.67E-005	1.56	235787_at	NA	NA
64	8.91E-005	1.42	212888_at	DICER1	dicer 1, ribonuclease type III
65	8.93E-005	1.93	1568593_a_at	NUDT16P	nudix (nucleoside diphosphate linked moiety X)-type motif 16 pseudogene
66	9.00E-005	1.92	226721_at	DPY19L4	dpy-19-like 4 (C. elegans)
67	9.02E-005	1.39	227159_at	GHDC	GH3 domain containing
68	9.14E-005	2.36	225982_at	UBTF	upstream binding transcription factor, RNA polymerase I
69	9.25E-005	1.62	220341_s_at	C5orf45	chromosome 5 open reading frame 45
70	9.51E-005	1.36	214501_s_at	H2AFY	H2A histone family, member Y
71	9.96E-005	7.6	226186_at	NA	NA
72	0.0001001	2.59	224946_s_at	CCDC115	coiled-coil domain containing 115
73	0.0001056	1.66	237059_at	NA	NA
74	0.0001076	3.11	38671_at	PLXND1	plexin D1
75	0.0001083	1.92	231912_s_at	DKFZP434B0335	DKFZP434B0335 protein
76	0.0001087	1.55	238492_at	NA	NA
77	0.0001088	2.05	228548_at	NA	NA
78	0.0001089	1.9	225757_s_at	CLMN	calmin (calponin-like, transmembrane)
79	0.0001093	1.15	203926_x_at	ATP5D	ATP synthase, H+ transporting, mitochondrial F1 complex, delta subunit
80	0.0001100	1.41	232520_s_at	NSFL1C	NSFL1 (p97) cofactor (p47)
81	0.0001101	2.06	238012_at	DPP7	dipeptidyl-peptidase 7
82	0.0001101	1.38	211101_x_at	LILRA2	leukocyte immunoglobulin-like receptor, subfamily A (with TM domain), member 2
83	0.0001109	1.35	210128_s_at	LTB4R	leukotriene B4 receptor
84	0.0001115	1.34	223393_s_at	TSHZ3	teashirt zinc finger homeobox 3
85	0.0001123	1.46	213628_at	CLCC1	chloride channel CLIC-like 1
86	0.0001152	1.27	214870_x_at	LOC100132540	similar to LOC339047 protein
87	0.0001171	2.41	221756_at	PIK3IP1	phosphoinositide-3-kinase interacting protein 1
88	0.0001171	1.62	222478_at	VPS36	vacuolar protein sorting 36 homolog (S. cerevisiae)
89	0.0001192	1.99	225719_s_at	MRPL55	mitochondrial ribosomal protein L55
90	0.0001206	1.87	212959_s_at	GNPTAB	N-acetylglucosamine-1-phosphate transferase, alpha and beta subunits
91	0.0001210	7.64	238520_at	TRERF1	transcriptional regulating factor 1
92	0.0001235	3.04	226974_at	NA	NA
93	0.0001269	2.06	225851_at	FNTB	farnesyltransferase, CAAX box, beta
94	0.0001288	2.04	224452_s_at	MGC12966	hypothetical protein LOC84792
95	0.0001306	1.46	226092_at	MPP5	membrane protein, palmitoylated 5 (MAGUK p55 subfamily member 5)
96	0.0001312	1.5	1558693_s_at	C1orf85	chromosome 1 open reading frame 85
97	0.0001336	1.19	204020_at	PURA	purine-rich element binding protein A
98	0.0001350	1.73	1555882_at	SPIN3	spindlin family, member 3
99	0.0001354	1.8	235532_at	PIGM	phosphatidylinositol glycan anchor biosynthesis, class M
100	0.0001371	2.61	1553111_a_at	KBTBD6	kelch repeat and BTB (POZ) domain containing 6
101	0.0001408	1.44	227868_at	LOC154761	hypothetical LOC154761
102	0.0001408	3.15	214058_at	MYCL1	v-myc myelocytomatosis viral oncogene homolog 1, lung carcinoma derived (avian)
103	0.0001409	2.65	212686_at	PPM1H	protein phosphatase 1H (PP2C domain containing)
104	0.0001414	1.61	218971_s_at	WDR91	WD repeat domain 91
105	0.0001418	2.07	225777_at	C9orf140	chromosome 9 open reading frame 140
106	0.0001426	1.92	225366_at	PGM2	phosphoglucomutase 2
107	0.0001451	1.74	238768_at	C2orf68	chromosome 2 open reading frame 68
108	0.0001454	1.49	218674_at	C5orf44	chromosome 5 open reading frame 44
109	0.0001462	1.26	218490_s_at	ZNF302	zinc finger protein 302
110	0.0001537	1.17	202704_at	TOB1	transducer of ERBB2, 1
111	0.0001561	1.69	218361_at	GOLPH3L	golgi phosphoprotein 3-like
112	0.0001565	1.32	222994_at	PRDX5	peroxiredoxin 5
113	0.0001590	2.19	209828_s_at	IL16	interleukin 16 (lymphocyte chemoattractant factor)
114	0.0001593	1.47	204611_s_at	PPP2R5B	protein phosphatase 2, regulatory subunit B@#$%&, beta isoform
115	0.0001593	1.48	227730_at	NA	NA
116	0.0001599	1.14	228605_at	NA	NA
117	0.0001646	1.54	202135_s_at	ACTR1B	ARP1 actin-related protein 1 homolog B, centractin beta (yeast)
118	0.0001669	1.73	226183_at	NA	NA
119	0.0001709	1.88	65472_at	C2orf68	chromosome 2 open reading frame 68
120	0.0001738	1.57	200098_s_at	ANAPC5	anaphase promoting complex subunit 5
121	0.0001748	1.37	218181_s_at	MAP4K4	mitogen-activated protein kinase kinase kinase kinase 4
122	0.0001762	2.81	238135_at	AGTRAP	angiotensin II receptor-associated protein
123	0.0001767	3.66	203386_at	TBC1D4	TBC1 domain family, member 4
124	0.0001784	2.06	213670_x_at	NSUN5B	NOL1/NOP2/Sun domain family, member 5B
125	0.0001787	1.69	212109_at	HN1L	hematological and neurological expressed 1-like
126	0.0001789	1.85	219968_at	ZNF589	zinc finger protein 589
127	0.0001799	2.25	211495_x_at	TNFSF13	tumor necrosis factor (ligand) superfamily, member 13
128	0.0001836	2.07	214177_s_at	PBXIP1	pre-B-cell leukemia homeobox interacting protein 1
129	0.0001843	1.64	1554085_at	DDX51	DEAD (Asp-Glu-Ala-Asp) box polypeptide 51
130	0.0001852	1.99	203271_s_at	UNC119	unc-119 homolog (C. elegans)
131	0.0001878	1.43	226072_at	FUK	fucokinase
132	0.0001878	3.32	212235_at	PLXND1	plexin D1
133	0.0001902	1.67	205658_s_at	SNAPC4	small nuclear RNA activating complex, polypeptide 4, 190kDa
134	0.0001983	1.84	218021_at	DHRS4	dehydrogenase/reductase (SDR family) member 4
135	0.0001987	1.76	229429_x_at	FAM91A2	family with sequence similarity 91, member A2
136	0.0001990	1.27	212429_s_at	GTF3C2	general transcription factor IIIC, polypeptide 2, beta 110kDa
137	0.0002007	1.81	226873_at	NA	NA
138	0.0002010	1.31	227801_at	TRIM59	tripartite motif-containing 59
139	0.0002032	1.36	227679_at	NA	NA
140	0.0002081	4.03	218326_s_at	LGR4	leucine-rich repeat-containing G protein-coupled receptor 4
141	0.0002087	1.93	235252_at	KSR1	kinase suppressor of ras 1
142	0.0002093	1.45	1558522_at	NA	NA
143	0.0002165	1.57	225396_at	NA	NA
144	0.0002167	1.4	206469_x_at	AKR7A3	aldo-keto reductase family 7, member A3 (aflatoxin aldehyde reductase)
145	0.0002197	1.79	1563549_a_at	ANO8	anoctamin 8
146	0.0002219	1.79	211576_s_at	SLC19A1	solute carrier family 19 (folate transporter), member 1
147	0.0002234	1.35	202846_s_at	PIGC	phosphatidylinositol glycan anchor biosynthesis, class C
148	0.0002246	2.17	232231_at	RUNX2	runt-related transcription factor 2
149	0.0002301	1.24	213480_at	VAMP4	vesicle-associated membrane protein 4
150	0.0002335	1.57	1558755_x_at	ZNF763	zinc finger protein 763
151	0.0002348	1.87	1557667_at	NA	NA
152	0.0002362	1.53	211036_x_at	ANAPC5	anaphase promoting complex subunit 5
153	0.0002367	2.73	203387_s_at	TBC1D4	TBC1 domain family, member 4
154	0.0002416	1.35	217896_s_at	NIP30	NEFA-interacting nuclear protein NIP30
155	0.0002420	2.35	212136_at	ATP2B4	ATPase, Ca++ transporting, plasma membrane 4
156	0.0002425	1.4	207618_s_at	BCS1L	BCS1-like (yeast)
157	0.0002450	1.85	239300_at	NA	NA
158	0.0002478	2.1	229497_at	ANKDD1A	ankyrin repeat and death domain containing 1A
159	0.0002509	1.29	214035_x_at	LOC399491	LOC399491 protein
160	0.0002517	1.66	229874_x_at	NA	NA
161	0.0002521	1.74	221264_s_at	LOC100128223	hypothetical protein LOC100128223
162	0.0002522	1.54	227630_at	NA	NA
163	0.0002531	2.4	226016_at	CD47	CD47 molecule
164	0.0002559	1.53	1558947_at	NA	NA
165	0.0002582	2.08	222664_at	KCTD15	potassium channel tetramerisation domain containing 15
166	0.0002606	4.49	207843_x_at	CYB5A	cytochrome b5 type A (microsomal)
167	0.0002633	2.1	218394_at	ROGDI	rogdi homolog (Drosophila)
168	0.0002634	1.86	214100_x_at	NSUN5B	NOL1/NOP2/Sun domain family, member 5B
169	0.0002641	1.4	212895_s_at	ABR	active BCR-related gene
170	0.0002648	2.01	227580_s_at	DKFZP434B0335	DKFZP434B0335 protein
171	0.0002678	2.59	227253_at	CP	ceruloplasmin (ferroxidase)
172	0.0002679	1.78	230917_at	NA	NA
173	0.0002686	1.69	200843_s_at	EPRS	glutamyl-prolyl-tRNA synthetase
174	0.0002686	2.67	227346_at	IKZF1	IKAROS family zinc finger 1 (Ikaros)
175	0.0002715	1.44	219095_at	LOC100137047-PLA2G4B	hypothetical protein LOC8681
176	0.0002762	1.68	229776_at	SLCO3A1	solute carrier organic anion transporter family, member 3A1
177	0.0002768	4.48	226436_at	RASSF4	Ras association (RalGDS/AF-6) domain family member 4
178	0.0002796	1.37	221951_at	TMEM80	transmembrane protein 80
179	0.0002809	1.49	228606_at	TM4SF19	transmembrane 4 L six family member 19
180	0.0002817	1.66	232535_at	NA	NA
181	0.0002826	1.28	1569597_at	NA	NA
182	0.0002831	1.77	1555845_at	NA	NA
183	0.0002835	2.77	205955_at	NA	NA
184	0.0002839	2.47	220137_at	FLJ20674	hypothetical protein FLJ20674
185	0.0002845	2.56	218459_at	TOR3A	torsin family 3, member A
186	0.0002870	1.73	238929_at	SFRS2B	splicing factor, arginine/serine-rich 2B
187	0.0002872	2.1	203317_at	PSD4	pleckstrin and Sec7 domain containing 4
188	0.0002969	1.32	238263_at	LOC285965	hypothetical protein LOC285965
189	0.0003005	2.64	235159_at	NA	NA
190	0.0003043	1.24	218388_at	PGLS	6-phosphogluconolactonase
191	0.0003057	1.3	206729_at	TNFRSF8	tumor necrosis factor receptor superfamily, member 8
192	0.0003121	1.28	231130_at	NA	NA
193	0.0003123	1.4	1552257_a_at	TTLL12	tubulin tyrosine ligase-like family, member 12
194	0.0003129	1.29	219175_s_at	SLC41A3	solute carrier family 41, member 3
195	0.0003162	1.41	204786_s_at	IFNAR2	interferon (alpha, beta and omega) receptor 2
196	0.0003176	1.47	225391_at	LOC93622	hypothetical LOC93622
197	0.0003216	1.35	227127_at	TMEM110	transmembrane protein 110
198	0.0003219	3.64	202341_s_at	TRIM2	tripartite motif-containing 2
199	0.0003243	2.09	210731_s_at	LGALS8	lectin, galactoside-binding, soluble, 8
200	0.0003251	1.77	213374_x_at	HIBCH	3-hydroxyisobutyryl-Coenzyme A hydrolase
201	0.0003260	1.38	200931_s_at	VCL	vinculin
202	0.0003264	1.45	230304_at	NA	NA
203	0.0003271	2.03	235195_at	FBXW2	F-box and WD repeat domain containing 2
204	0.0003290	4.23	215726_s_at	CYB5A	cytochrome b5 type A (microsomal)
205	0.0003300	2.01	242794_at	MAML3	mastermind-like 3 (Drosophila)
206	0.0003304	2.18	225961_at	KLHDC5	kelch domain containing 5
207	0.0003309	1.48	212556_at	SCRIB	scribbled homolog (Drosophila)
208	0.0003322	2.66	220494_s_at	NA	NA
209	0.0003337	2.38	242297_at	RREB1	ras responsive element binding protein 1
210	0.0003349	1.9	228771_at	ADRBK2	adrenergic, beta, receptor kinase 2
211	0.0003390	1.19	227465_at	KIAA0892	KIAA0892
212	0.0003390	1.37	228667_at	AGPAT4	1-acylglycerol-3-phosphate O-acyltransferase 4 (lysophosphatidic acid acyltransferase, delta)
213	0.0003408	1.72	202161_at	PKN1	protein kinase N1
214	0.0003417	1.32	AFFX-LysX-M_at	NA	NA
215	0.0003429	1.57	223314_at	TSPAN14	tetraspanin 14
216	0.0003438	2.51	204610_s_at	CCDC85B	coiled-coil domain containing 85B
217	0.0003438	2.03	218027_at	MRPL15	mitochondrial ribosomal protein L15
218	0.0003450	2.45	204718_at	EPHB6	EPH receptor B6
219	0.0003454	2.06	227313_at	CNPY4	canopy 4 homolog (zebrafish)
220	0.0003454	1.52	228600_x_at	C7orf46	chromosome 7 open reading frame 46
221	0.0003472	1.28	226335_at	RPS6KA3	ribosomal protein S6 kinase, 90kDa, polypeptide 3
222	0.0003481	1.48	219147_s_at	C9orf95	chromosome 9 open reading frame 95
223	0.0003492	1.41	219801_at	ZNF34	zinc finger protein 34
224	0.0003534	1.37	224865_at	FAR1	fatty acyl CoA reductase 1
225	0.0003534	1.25	209450_at	OSGEP	O-sialoglycoprotein endopeptidase
226	0.0003535	1.8	239016_at	NA	NA
227	0.0003567	1.44	228670_at	TEP1	telomerase-associated protein 1
228	0.0003600	1.87	210580_x_at	SULT1A3	sulfotransferase family, cytosolic, 1A, phenol-preferring, member 3
229	0.0003607	1.38	213945_s_at	NUP210	nucleoporin 210kDa
230	0.0003644	1.32	218505_at	WDR59	WD repeat domain 59
231	0.0003661	2.32	230343_at	NA	NA
232	0.0003663	2.08	230888_at	WDR91	WD repeat domain 91
233	0.0003691	1.87	226368_at	CHST11	carbohydrate (chondroitin 4) sulfotransferase 11
234	0.0003691	1.67	213364_s_at	SNX1	sorting nexin 1
235	0.0003706	2.17	213626_at	CBR4	carbonyl reductase 4
236	0.0003712	1.45	AFFX-PheX-3_at	NA	NA
237	0.0003730	1.59	206567_s_at	PHF20	PHD finger protein 20
238	0.0003737	1.49	221090_s_at	OGFOD1	2-oxoglutarate and iron-dependent oxygenase domain containing 1
239	0.0003762	1.33	44040_at	FBXO41	F-box protein 41
240	0.0003796	2.03	226238_at	MCEE	methylmalonyl CoA epimerase
241	0.0003812	1.45	204562_at	IRF4	interferon regulatory factor 4
242	0.0003827	1.46	226241_s_at	MRPL52	mitochondrial ribosomal protein L52
243	0.0003831	1.46	220178_at	C19orf28	chromosome 19 open reading frame 28
244	0.0003841	1.31	209263_x_at	TSPAN4	tetraspanin 4
245	0.0003893	1.45	232228_at	ZNF530	zinc finger protein 530
246	0.0003907	2.04	208760_at	UBE2I	ubiquitin-conjugating enzyme E2I (UBC9 homolog, yeast)
247	0.0003909	1.18	224562_at	WASF2	WAS protein family, member 2
248	0.0003923	1.63	213485_s_at	ABCC10	ATP-binding cassette, sub-family C (CFTR/MRP), member 10
249	0.0003984	1.13	202942_at	ETFB	electron-transfer-flavoprotein, beta polypeptide
250	0.0004011	1.54	AFFX-LysX-3_at	NA	NA
251	0.0004028	1.79	212135_s_at	ATP2B4	ATPase, Ca++ transporting, plasma membrane 4
252	0.0004062	1.24	217828_at	SLTM	SAFB-like, transcription modulator
253	0.0004150	2.05	212875_s_at	C2CD2	C2 calcium-dependent domain containing 2
254	0.0004182	2.92	1557411_s_at	SLC25A43	solute carrier family 25, member 43
255	0.0004259	2.11	227117_at	NA	NA
256	0.0004308	1.57	207124_s_at	GNB5	guanine nucleotide binding protein (G protein), beta 5
257	0.0004325	1.6	227607_at	STAMBPL1	STAM binding protein-like 1
258	0.0004326	1.25	204538_x_at	NPIP	nuclear pore complex interacting protein
259	0.0004339	2.03	244619_at	BCL10	B-cell CLL/lymphoma 10
260	0.0004343	1.38	223239_at	C14orf129	chromosome 14 open reading frame 129
261	0.0004347	1.58	201087_at	PXN	paxillin
262	0.0004367	1.8	219149_x_at	DBR1	debranching enzyme homolog 1 (S. cerevisiae)
263	0.0004371	1.88	229905_at	RAP1GDS1	RAP1, GTP-GDP dissociation stimulator 1
264	0.0004382	1.61	222111_at	NA	NA
265	0.0004389	2.71	235052_at	ZNF792	zinc finger protein 792
266	0.0004422	1.62	225748_at	LTV1	LTV1 homolog (S. cerevisiae)
267	0.0004451	1.37	241741_at	CRLS1	cardiolipin synthase 1
268	0.0004463	1.46	221504_s_at	ATP6V1H	ATPase, H+ transporting, lysosomal 50/57kDa, V1 subunit H
269	0.0004468	1.98	213448_at	NA	NA
270	0.0004483	1.15	201949_x_at	CAPZB	capping protein (actin filament) muscle Z-line, beta
271	0.0004501	2.15	234295_at	DBR1	debranching enzyme homolog 1 (S. cerevisiae)
272	0.0004505	1.72	217608_at	SFRS12IP1	SFRS12-interacting protein 1
273	0.0004518	1.34	215737_x_at	USF2	upstream transcription factor 2, c-fos interacting
274	0.0004529	1.47	215873_x_at	ABCC10	ATP-binding cassette, sub-family C (CFTR/MRP), member 10
275	0.0004530	2.34	1552256_a_at	SCARB1	scavenger receptor class B, member 1
276	0.0004546	2.47	208657_s_at	9-Sep	septin 9
277	0.0004555	2.08	228096_at	C1orf151	chromosome 1 open reading frame 151
278	0.0004560	1.75	222471_s_at	KCMF1	potassium channel modulatory factor 1
279	0.0004590	1.55	48808_at	DHFR	dihydrofolate reductase
280	0.0004608	3.5	227228_s_at	CCDC88C	coiled-coil domain containing 88C
281	0.0004636	1.94	1558445_at	NA	NA
282	0.0004641	1.13	205540_s_at	RRAGB	Ras-related GTP binding B
283	0.0004670	1.48	227239_at	FAM126A	family with sequence similarity 126, member A
284	0.0004673	1.72	220246_at	CAMK1D	calcium/calmodulin-dependent protein kinase ID
285	0.0004677	3.38	226478_at	NA	NA
286	0.0004700	1.41	230235_at	NA	NA
287	0.0004709	1.22	220750_s_at	LEPRE1	leucine proline-enriched proteoglycan (leprecan) 1
288	0.0004727	2.69	223455_at	TCHP	trichoplein, keratin filament binding
289	0.0004736	1.31	238552_at	NA	NA
290	0.0004739	1.47	200827_at	PLOD1	procollagen-lysine 1, 2-oxoglutarate 5-dioxygenase 1
291	0.0004751	1.28	227710_s_at	NA	NA
292	0.0004785	4.77	236798_at	NA	NA
293	0.0004788	1.88	242824_at	NA	NA
294	0.0004793	1.08	215846_at	NA	NA
295	0.0004795	1.92	211385_x_at	SULT1A2	sulfotransferase family, cytosolic, 1A, phenol-preferring, member 2
296	0.0004800	1.38	226358_at	LOC145842	hypothetical protein LOC145842
297	0.0004832	1.95	213534_s_at	PASK	PAS domain containing serine/threonine kinase
298	0.0004841	5.65	205698_s_at	MAP2K6	mitogen-activated protein kinase kinase 6
299	0.0004850	1.53	222661_at	AGGF1	angiogenic factor with G patch and FHA domains 1
300	0.0004855	1.43	212036_s_at	PNN	pinin, desmosome associated protein
301	0.0004858	1.6	244534_at	NA	NA
302	0.0004876	1.58	1555751_a_at	GEMIN7	gem (nuclear organelle) associated protein 7
303	0.0004879	1.87	203063_at	PPM1F	protein phosphatase 1F (PP2C domain containing)
304	0.0004973	1.16	205922_at	VNN2	vanin 2
305	0.0004975	1.23	202797_at	SACM1L	SAC1 suppressor of actin mutations 1-like (yeast)
306	0.0005017	2.46	202826_at	SPINT1	serine peptidase inhibitor, Kunitz type 1
307	0.0005059	1.73	226073_at	TMEM218	transmembrane protein 218
308	0.0005077	1.55	238523_at	KLHL36	kelch-like 36 (Drosophila)
309	0.0005080	1.78	231843_at	DDX55	DEAD (Asp-Glu-Ala-Asp) box polypeptide 55
310	0.0005094	2.26	219714_s_at	CACNA2D3	calcium channel, voltage-dependent, alpha 2/delta subunit 3
311	0.0005097	2.02	229202_at	NA	NA
312	0.0005114	3.54	209048_s_at	ZMYND8	zinc finger, MYND-type containing 8
313	0.0005132	1.64	218473_s_at	GLT25D1	glycosyltransferase 25 domain containing 1
314	0.0005172	1.71	65493_at	HEATR6	HEAT repeat containing 6
315	0.0005179	2.03	236194_at	NA	NA
316	0.0005179	2.28	226531_at	ORAI1	ORAI calcium release-activated calcium modulator 1
317	0.0005201	1.58	219351_at	TRAPPC2	trafficking protein particle complex 2
318	0.0005244	1.26	220036_s_at	LMBR1L	limb region 1 homolog (mouse)-like
319	0.0005321	4.22	217974_at	TM7SF3	transmembrane 7 superfamily member 3
320	0.0005335	1.26	211759_x_at	TBCB	tubulin folding cofactor B
321	0.0005359	1.4	242155_x_at	NA	NA
322	0.0005397	2	209377_s_at	HMGN3	high mobility group nucleosomal binding domain 3
323	0.0005401	2.12	230653_at	LOC100132218	hypothetical protein LOC100132218
324	0.0005504	1.77	224708_at	KIAA2013	KIAA2013
325	0.0005504	1.9	204000_at	GNB5	guanine nucleotide binding protein (G protein), beta 5
326	0.0005559	1.32	244346_at	NA	NA
327	0.0005568	1.9	225108_at	AGPS	alkylglycerone phosphate synthase
328	0.0005599	1.85	236626_at	NA	NA
329	0.0005615	1.85	228314_at	LRRC8C	leucine rich repeat containing 8 family, member C
330	0.0005636	1.46	1558754_at	ZNF763	zinc finger protein 763
331	0.0005650	1.42	226155_at	FAM160B1	family with sequence similarity 160, member B1
332	0.0005679	1.72	229705_at	NA	NA
333	0.0005686	4.21	228891_at	C9orf164	chromosome 9 open reading frame 164
334	0.0005708	1.49	225146_at	C9orf25	chromosome 9 open reading frame 25
335	0.0005724	1.86	219817_at	C12orf47	chromosome 12 open reading frame 47
336	0.0005724	1.58	235610_at	ALKBH8	alkB, alkylation repair homolog 8 (E. coli)
337	0.0005728	1.59	217949_s_at	VKORC1	vitamin K epoxide reductase complex, subunit 1
338	0.0005746	2.14	222858_s_at	DAPP1	dual adaptor of phosphotyrosine and 3-phosphoinositides
339	0.0005748	1.17	223049_at	GRB2	growth factor receptor-bound protein 2
340	0.0005792	1.45	212987_at	FBXO9	F-box protein 9
341	0.0005793	1.42	209903_s_at	ATR	ataxia telangiectasia and Rad3 related
342	0.0005805	1.45	201067_at	PSMC2	proteasome (prosome, macropain) 26S subunit, ATPase, 2
343	0.0005806	1.37	201076_at	NHP2L1	NHP2 non-histone chromosome protein 2-like 1 (S. cerevisiae)
344	0.0005811	1.28	236804_at	NA	NA
345	0.0005820	1.27	234107_s_at	DTD1	D-tyrosyl-tRNA deacylase 1 homolog (S. cerevisiae)
346	0.0005858	2.04	1553987_at	C12orf47	chromosome 12 open reading frame 47
347	0.0005877	1.42	226679_at	SLC26A11	solute carrier family 26, member 11
348	0.0005893	1.7	1554608_at	TGOLN2	trans-golgi network protein 2
349	0.0005911	1.65	219256_s_at	SH3TC1	SH3 domain and tetratricopeptide repeats 1
350	0.0005950	1.46	232369_at	NA	NA
351	0.0005992	1.39	243750_x_at	C21orf70	chromosome 21 open reading frame 70
352	0.0006007	2.4	219759_at	ERAP2	endoplasmic reticulum aminopeptidase 2
353	0.0006025	1.24	203981_s_at	ABCD4	ATP-binding cassette, sub-family D (ALD), member 4
354	0.0006028	1.43	202428_x_at	DBI	diazepam binding inhibitor (GABA receptor modulator, acyl-Coenzyme A binding protein)
355	0.0006039	1.54	212886_at	CCDC69	coiled-coil domain containing 69
356	0.0006041	2.12	221878_at	C2orf68	chromosome 2 open reading frame 68
357	0.0006052	1.91	202039_at	TIAF1	TGFB1-induced anti-apoptotic factor 1
358	0.0006058	2.8	40472_at	LPCAT4	lysophosphatidylcholine acyltransferase 4
359	0.0006135	1.48	217751_at	GSTK1	glutathione S-transferase kappa 1
360	0.0006135	1.84	228303_at	GALNT6	UDP-N-acetyl-alpha-D-galactosamine:polypeptide N-acetylgalactosaminyltransferase 6 (GalNAc-T6)
361	0.0006147	1.44	202172_at	VEZF1	vascular endothelial zinc finger 1
362	0.0006167	2.09	1558692_at	C1orf85	chromosome 1 open reading frame 85
363	0.0006190	1.89	207122_x_at	SULT1A2	sulfotransferase family, cytosolic, 1A, phenol-preferring, member 2
364	0.0006205	1.49	1560874_at	FLJ33046	hypothetical gene supported by AK057608
365	0.0006228	1.97	212473_s_at	MICAL2	microtubule associated monoxygenase, calponin and LIM domain containing 2
366	0.0006241	1.67	225409_at	C2orf64	chromosome 2 open reading frame 64
367	0.0006246	1.97	203615_x_at	SULT1A1	sulfotransferase family, cytosolic, 1A, phenol-preferring, member 1
368	0.0006275	1.4	224724_at	SULF2	sulfatase 2
369	0.0006277	1.47	225022_at	GOPC	golgi associated PDZ and coiled-coil motif containing
370	0.0006282	1.18	214879_x_at	USF2	upstream transcription factor 2, c-fos interacting
371	0.0006311	1.43	222843_at	FIGNL1	fidgetin-like 1
372	0.0006313	2.06	210136_at	MBP	myelin basic protein
373	0.0006322	2.27	229512_at	FAM120C	family with sequence similarity 120C
374	0.0006326	1.2	209017_s_at	LONP1	lon peptidase 1, mitochondrial
375	0.0006333	1.69	237926_s_at	NA	NA
376	0.0006351	1.44	222294_s_at	RAB27A	RAB27A, member RAS oncogene family
377	0.0006411	3.49	210986_s_at	TPM1	tropomyosin 1 (alpha)
378	0.0006490	1.28	209932_s_at	DUT	deoxyuridine triphosphatase
379	0.0006513	1.28	227656_at	C6orf70	chromosome 6 open reading frame 70
380	0.0006514	1.63	228131_at	ERCC1	excision repair cross-complementing rodent repair deficiency, complementation group 1 (includes overlapping antisense sequence)
381	0.0006519	1.11	212848_s_at	C9orf3	chromosome 9 open reading frame 3
382	0.0006526	2.3	1552540_s_at	IQCD	IQ motif containing D
383	0.0006530	2.09	239698_at	NA	NA
384	0.0006586	1.62	1553102_a_at	CCDC69	coiled-coil domain containing 69
385	0.0006622	1.77	228542_at	MRS2	MRS2 magnesium homeostasis factor homolog (S. cerevisiae)
386	0.0006623	1.37	208956_x_at	DUT	deoxyuridine triphosphatase
387	0.0006635	2.14	223528_s_at	METT11D1	methyltransferase 11 domain containing 1
388	0.0006636	1.38	201234_at	ILK	integrin-linked kinase
389	0.0006637	1.57	228694_at	NA	NA
390	0.0006659	1.36	225136_at	PLEKHA2	pleckstrin homology domain containing, family A (phosphoinositide binding specific) member 2
391	0.0006723	1.55	212567_s_at	MAP4	microtubule-associated protein 4
392	0.0006726	1.52	219549_s_at	RTN3	reticulon 3
393	0.0006730	1.89	232681_at	NA	NA
394	0.0006742	2.21	219627_at	ZNF767	zinc finger family member 767
395	0.0006762	2.7	231449_at	NA	NA
396	0.0006771	1.58	239035_at	MTHFR	5,10-methylenetetrahydrofolate reductase (NADPH)
397	0.0006773	1.39	205256_at	ZBTB39	zinc finger and BTB domain containing 39
398	0.0006786	1.51	205945_at	IL6R	interleukin 6 receptor
399	0.0006802	4.26	230032_at	OSGEPL1	O-sialoglycoprotein endopeptidase-like 1
400	0.0006837	1.56	225888_at	C12orf30	chromosome 12 open reading frame 30
401	0.0006840	1.35	227767_at	CSNK1G3	casein kinase 1, gamma 3
402	0.0006879	1.76	205060_at	PARG	poly (ADP-ribose) glycohydrolase
403	0.0006921	1.37	239730_at	DGCR14	DiGeorge syndrome critical region gene 14
404	0.0006924	1.58	201029_s_at	CD99	CD99 molecule
405	0.0006928	1.63	211709_s_at	CLEC11A	C-type lectin domain family 11, member A
406	0.0006952	1.95	201985_at	KIAA0196	KIAA0196
407	0.0006964	2.17	204995_at	CDK5R1	cyclin-dependent kinase 5, regulatory subunit 1 (p35)
408	0.0007029	1.52	217521_at	NA	NA
409	0.0007045	1.35	1558184_s_at	ZNF17	zinc finger protein 17
410	0.0007099	1.24	218167_at	AMZ2	archaelysin family metallopeptidase 2
411	0.0007119	1.52	226712_at	SSR1	signal sequence receptor, alpha
412	0.0007129	1.22	238668_at	NA	NA
413	0.0007138	1.16	221651_x_at	IGKC	immunoglobulin kappa constant
414	0.0007143	1.85	64064_at	GIMAP5	GTPase, IMAP family member 5
415	0.0007160	1.24	234734_s_at	TNRC6A	trinucleotide repeat containing 6A
416	0.0007165	1.34	213582_at	ATP11A	ATPase, class VI, type 11A
417	0.0007176	1.34	226165_at	C8orf59	chromosome 8 open reading frame 59
418	0.0007186	2.61	205565_s_at	FXN	frataxin
419	0.0007225	1.21	220251_at	C1orf107	chromosome 1 open reading frame 107
420	0.0007231	2.16	225980_at	C14orf43	chromosome 14 open reading frame 43
421	0.0007247	1.69	238379_x_at	NA	NA
422	0.0007266	1.72	1559034_at	SIRPB2	signal-regulatory protein beta 2
423	0.0007273	1.21	201053_s_at	PSMF1	proteasome (prosome, macropain) inhibitor subunit 1 (PI31)
424	0.0007318	1.1	40225_at	GAK	cyclin G associated kinase
425	0.0007329	2.14	209729_at	GAS2L1	growth arrest-specific 2 like 1
426	0.0007344	1.55	221027_s_at	PLA2G12A	phospholipase A2, group XIIA
427	0.0007348	1.28	209724_s_at	ZFP161	zinc finger protein 161 homolog (mouse)
428	0.0007380	1.4	214494_s_at	SPG7	spastic paraplegia 7 (pure and complicated autosomal recessive)
429	0.0007392	1.58	205131_x_at	CLEC11A	C-type lectin domain family 11, member A
430	0.0007393	2.07	204019_s_at	SH3YL1	SH3 domain containing, Ysc84-like 1 (S. cerevisiae)
431	0.0007417	1.42	214861_at	JMJD2C	jumonji domain containing 2C
432	0.0007421	1.69	242965_at	NA	NA
433	0.0007485	1.99	228167_at	KLHL6	kelch-like 6 (Drosophila)
434	0.0007547	2.15	209269_s_at	SYK	spleen tyrosine kinase
435	0.0007563	1.5	244663_at	NA	NA
436	0.0007563	2.14	203802_x_at	NSUN5	NOL1/NOP2/Sun domain family, member 5
437	0.0007578	1.62	242108_at	NA	NA
438	0.0007655	1.46	205632_s_at	PIP5K1B	phosphatidylinositol-4-phosphate 5-kinase, type I, beta
439	0.0007691	2.28	238604_at	NA	NA
440	0.0007694	1.25	219084_at	NSD1	nuclear receptor binding SET domain protein 1
441	0.0007712	1.4	223716_s_at	ZRANB2	zinc finger, RAN-binding domain containing 2
442	0.0007728	1.82	209760_at	KIAA0922	KIAA0922
443	0.0007796	1.29	214437_s_at	SHMT2	serine hydroxymethyltransferase 2 (mitochondrial)
444	0.0007836	1.46	224704_at	TNRC6A	trinucleotide repeat containing 6A
445	0.0007841	2.01	223339_at	ATPIF1	ATPase inhibitory factor 1
446	0.0007848	1.59	222622_at	PGP	phosphoglycolate phosphatase
447	0.0007851	1.62	218231_at	NAGK	N-acetylglucosamine kinase
448	0.0007878	1.79	1554544_a_at	MBP	myelin basic protein
449	0.0007894	2.2	1554250_s_at	TRIM73	tripartite motif-containing 73
450	0.0007896	2.19	216199_s_at	MAP3K4	mitogen-activated protein kinase kinase kinase 4
451	0.0007925	1.3	206881_s_at	LILRA3	leukocyte immunoglobulin-like receptor, subfamily A (without TM domain), member 3
452	0.0007976	1.65	226716_at	PRR12	proline rich 12
453	0.0007989	1.67	202534_x_at	DHFR	dihydrofolate reductase
454	0.0007995	2.43	202369_s_at	TRAM2	translocation associated membrane protein 2
455	0.0008009	2.59	218112_at	MRPS34	mitochondrial ribosomal protein S34
456	0.0008035	1.48	230925_at	APBB1IP	amyloid beta (A4) precursor protein-binding, family B, member 1 interacting protein
457	0.0008086	1.16	213027_at	TROVE2	TROVE domain family, member 2
458	0.0008124	2.99	1562289_at	NA	NA
459	0.0008148	1.41	202615_at	GNAQ	guanine nucleotide binding protein (G protein), q polypeptide
460	0.0008150	1.71	219151_s_at	RABL2B	RAB, member of RAS oncogene family-like 2B
461	0.0008158	2.1	1559214_at	NA	NA
462	0.0008161	1.84	203711_s_at	HIBCH	3-hydroxyisobutyryl-Coenzyme A hydrolase
463	0.0008187	1.87	233955_x_at	CXXC5	CXXC finger 5
464	0.0008205	1.26	201804_x_at	TBCB	tubulin folding cofactor B
465	0.0008207	1.44	211100_x_at	LILRA2	leukocyte immunoglobulin-like receptor, subfamily A (with TM domain), member 2
466	0.0008229	5.13	212757_s_at	CAMK2G	calcium/calmodulin-dependent protein kinase (CaM kinase) II gamma
467	0.0008232	1.76	214202_at	NA	NA
468	0.0008255	2.01	221746_at	UBL4A	ubiquitin-like 4A
469	0.0008277	1.35	1560587_s_at	PRDX5	peroxiredoxin 5
470	0.0008278	1.41	211070_x_at	DBI	diazepam binding inhibitor (GABA receptor modulator, acyl-Coenzyme A binding protein)
471	0.0008279	1.53	242887_at	KCMF1	potassium channel modulatory factor 1
472	0.0008283	1.29	206200_s_at	ANXA11	annexin A11
473	0.0008318	1.72	203607_at	INPP5F	inositol polyphosphate-5-phosphatase F
474	0.0008344	1.9	205282_at	LRP8	low density lipoprotein receptor-related protein 8, apolipoprotein e receptor
475	0.0008347	1.42	209566_at	INSIG2	insulin induced gene 2
476	0.0008361	1.54	223306_at	EBPL	emopamil binding protein-like
477	0.0008444	3.9	210166_at	TLR5	toll-like receptor 5
478	0.0008451	1.44	225050_at	ZNF512	zinc finger protein 512
479	0.0008463	1.9	226480_at	NA	NA
480	0.0008510	1.22	211152_s_at	HTRA2	HtrA serine peptidase 2
481	0.0008526	1.9	222603_at	ERMP1	endoplasmic reticulum metallopeptidase 1
482	0.0008590	1.42	226078_at	RPUSD1	RNA pseudouridylate synthase domain containing 1
483	0.0008602	1.89	203409_at	DDB2	damage-specific DNA binding protein 2, 48kDa
484	0.0008607	1.79	222996_s_at	CXXC5	CXXC finger 5
485	0.0008619	1.42	229597_s_at	WDFY4	WDFY family member 4
486	0.0008625	1.21	209420_s_at	SMPD1	sphingomyelin phosphodiesterase 1, acid lysosomal
487	0.0008648	2.16	213333_at	MDH2	malate dehydrogenase 2, NAD (mitochondrial)
488	0.0008654	1.57	232524_x_at	ANAPC4	anaphase promoting complex subunit 4
489	0.0008674	1.63	238058_at	FLJ27365	FLJ27365 protein
490	0.0008682	2.88	212660_at	PHF15	PHD finger protein 15
491	0.0008701	2.47	209197_at	SYT11	synaptotagmin XI
492	0.0008755	1.49	200875_s_at	NOL5A	nucleolar protein 5A (56kDa with KKE/D repeat)
493	0.0008868	5.04	207008_at	IL8RB	interleukin 8 receptor, beta
494	0.0008925	1.33	233694_at	HSPA1L	heat shock 70kDa protein 1-like
495	0.0008958	1.35	217957_at	C16orf80	chromosome 16 open reading frame 80
496	0.0008961	1.64	228070_at	PPP2R5E	protein phosphatase 2, regulatory subunit B@#$%&, epsilon isoform
497	0.0008967	1.56	225997_at	MOBKL1A	MOB1, Mps One Binder kinase activator-like 1A (yeast)
498	0.0009020	1.98	65438_at	KIAA1609	KIAA1609
499	0.0009056	1.82	218921_at	SIGIRR	single immunoglobulin and toll-interleukin 1 receptor (TIR) domain
500	0.0009103	1.06	240001_at	NA	NA
501	0.0009114	1.51	227533_at	NA	NA
502	0.0009136	1.47	226333_at	IL6R	interleukin 6 receptor
503	0.0009154	1.68	1562249_at	LOC285965	hypothetical protein LOC285965
504	0.0009189	3.86	204301_at	KBTBD11	kelch repeat and BTB (POZ) domain containing 11
505	0.0009197	1.67	213296_at	RER1	RER1 retention in endoplasmic reticulum 1 homolog (S. cerevisiae)
506	0.0009228	1.32	224688_at	C7orf42	chromosome 7 open reading frame 42
507	0.0009245	1.91	221843_s_at	KIAA1609	KIAA1609
508	0.0009272	1.24	1569808_at	NA	NA
509	0.0009308	5.94	38290_at	RGS14	regulator of G-protein signaling 14
510	0.0009357	3.85	226820_at	ZNF362	zinc finger protein 362
511	0.0009370	1.35	241344_at	NA	NA
512	0.0009378	1.73	228512_at	PTCD3	Pentatricopeptide repeat domain 3
513	0.0009417	1.63	210830_s_at	PON2	paraoxonase 2
514	0.0009436	1.44	219493_at	SHCBP1	SHC SH2-domain binding protein 1
515	0.0009471	1.38	230122_at	MLLT10	myeloid/lymphoid or mixed-lineage leukemia (trithorax homolog, Drosophila); translocated to, 10
516	0.0009526	1.67	218380_at	NLRP1	NLR family, pyrin domain containing 1
517	0.0009562	1.42	202200_s_at	SRPK1	SFRS protein kinase 1
518	0.0009575	1.63	202741_at	PRKACB	protein kinase, cAMP-dependent, catalytic, beta
519	0.0009592	1.43	228869_at	NA	NA
520	0.0009614	1.25	224252_s_at	FXYD5	FXYD domain containing ion transport regulator 5
521	0.0009626	1.45	221488_s_at	CUTA	cutA divalent cation tolerance homolog (E. coli)
522	0.0009634	1.7	244687_at	DBT	dihydrolipoamide branched chain transacylase E2
523	0.0009679	1.17	209445_x_at	C7orf44	chromosome 7 open reading frame 44
524	0.0009703	1.14	244537_at	NA	NA
525	0.0009717	1.7	226104_at	RNF170	ring finger protein 170
526	0.0009730	1.31	205240_at	GPSM2	G-protein signaling modulator 2 (AGS3-like, C. elegans)
527	0.0009732	1.73	200766_at	CTSD	cathepsin D
528	0.0009734	1.41	231844_at	MGC27345	hypothetical protein MGC27345
529	0.0009738	1.29	202634_at	POLR2K	polymerase (RNA) II (DNA directed) polypeptide K, 7.0kDa
530	0.0009760	1.58	224938_at	NUFIP2	nuclear fragile X mental retardation protein interacting protein 2
531	0.0009764	2.26	204089_x_at	MAP3K4	mitogen-activated protein kinase kinase kinase 4
532	0.0009776	1.93	211985_s_at	CALM1	calmodulin 1 (phosphorylase kinase, delta)
533	0.0009778	2.65	213280_at	GARNL4	GTPase activating Rap/RanGAP domain-like 4
534	0.0009805	1.48	236016_at	NA	NA
535	0.0009828	1.84	217394_at	NA	NA
536	0.0009900	1.58	201030_x_at	LDHB	lactate dehydrogenase B
537	0.0009925	1.81	227379_at	MBOAT1	membrane bound O-acyltransferase domain containing 1
538	0.0009997	-1.19	236663_at	NA	NA
539	0.0009983	-1.6	219766_at	B9D2	B9 protein domain 2
540	0.0009949	-1.71	204157_s_at	KIAA0999	KIAA0999 protein
541	0.0009891	-1.23	214060_at	SSBP1	single-stranded DNA binding protein 1
542	0.0009868	-1.47	226276_at	TMEM167A	transmembrane protein 167A
543	0.0009825	-1.75	203596_s_at	IFIT5	interferon-induced protein with tetratricopeptide repeats 5
544	0.0009805	-1.64	226310_at	RICTOR	rapamycin-insensitive companion of mTOR
545	0.0009772	-2.29	218986_s_at	DDX60	DEAD (Asp-Glu-Ala-Asp) box polypeptide 60
546	0.0009758	-1.52	217618_x_at	HUS1	HUS1 checkpoint homolog (S. pombe)
547	0.0009746	-2.4	219017_at	ETNK1	ethanolamine kinase 1
548	0.0009745	-1.24	201957_at	PPP1R12B	protein phosphatase 1, regulatory (inhibitor) subunit 12B
549	0.0009737	-1.26	240887_at	NA	NA
550	0.0009681	-1.33	209514_s_at	RAB27A	RAB27A, member RAS oncogene family
551	0.0009661	-2.54	219026_s_at	RASAL2	RAS protein activator like 2
552	0.0009592	-1.49	211395_x_at	FCGR2C	Fc fragment of IgG, low affinity IIc, receptor for (CD32)
553	0.0009568	-3.72	205126_at	VRK2	vaccinia related kinase 2
554	0.0009560	-1.22	1556514_at	LOC338809	hypothetical protein LOC338809
555	0.0009506	-2.66	206991_s_at	CCR5	chemokine (C-C motif) receptor 5
556	0.0009500	-1.47	212840_at	UBXN7	UBX domain protein 7
557	0.0009443	-1.25	244496_at	NA	NA
558	0.0009406	-1.21	236108_at	KIAA1632	KIAA1632
559	0.0009366	-1.2	203654_s_at	COIL	coilin
560	0.0009338	-2.95	236156_at	LIPA	lipase A, lysosomal acid, cholesterol esterase
561	0.0009309	-1.3	212462_at	MYST4	MYST histone acetyltransferase (monocytic leukemia) 4
562	0.0009278	-2.58	219403_s_at	HPSE	heparanase
563	0.0009220	-1.58	1554747_a_at	2-Sep	septin 2
564	0.0009134	-1.08	203291_at	CNOT4	CCR4-NOT transcription complex, subunit 4
565	0.0009077	-1.25	243772_at	SDCCAG8	serologically defined colon cancer antigen 8
566	0.0009071	-1.7	201656_at	ITGA6	integrin, alpha 6
567	0.0009063	-3.06	201325_s_at	EMP1	epithelial membrane protein 1
568	0.0009031	-1.27	209531_at	GSTZ1	glutathione transferase zeta 1
569	0.0008974	-1.42	208779_x_at	DDR1	discoidin domain receptor tyrosine kinase 1
570	0.0008959	-1.19	242654_at	FANCC	Fanconi anemia, complementation group C
571	0.0008945	-1.16	220386_s_at	EML4	echinoderm microtubule associated protein like 4
572	0.0008922	-1.32	227003_at	RAB28	RAB28, member RAS oncogene family
573	0.0008910	-3.14	224009_x_at	DHRS9	dehydrogenase/reductase (SDR family) member 9
574	0.0008877	-1.67	32069_at	N4BP1	NEDD4 binding protein 1
575	0.0008870	-1.29	232141_at	U2AF1	U2 small nuclear RNA auxiliary factor 1
576	0.0008761	-1.35	240468_at	NA	NA
577	0.0008698	-1.32	204367_at	SP2	Sp2 transcription factor
578	0.0008616	-1.87	225076_s_at	ZNFX1	zinc finger, NFX1-type containing 1
579	0.0008599	-1.25	225056_at	SIPA1L2	signal-induced proliferation-associated 1 like 2
580	0.0008580	-1.13	207070_at	RGR	retinal G protein coupled receptor
581	0.0008574	-1.28	217129_at	NA	NA
582	0.0008537	-1.22	225268_at	KPNA4	karyopherin alpha 4 (importin alpha 3)
583	0.0008463	-1.07	232295_at	GFM1	G elongation factor, mitochondrial 1
584	0.0008408	-1.33	211975_at	ARFGAP2	ADP-ribosylation factor GTPase activating protein 2
585	0.0008385	-1.24	244625_at	NA	NA
586	0.0008384	-1.49	202083_s_at	SEC14L1	SEC14-like 1 (S. cerevisiae)
587	0.0008274	-1.34	232987_at	ARL17	ADP-ribosylation factor-like 17
588	0.0008198	-1.33	1570541_s_at	NA	NA
589	0.0008196	-2.91	201324_at	EMP1	epithelial membrane protein 1
590	0.0008172	-1.42	222408_s_at	YPEL5	yippee-like 5 (Drosophila)
591	0.0008164	-1.3	201585_s_at	SFPQ	splicing factor proline/glutamine-rich (polypyrimidine tract binding protein associated)
592	0.0008160	-1.35	243492_at	THEM4	thioesterase superfamily member 4
593	0.0008023	-1.53	222537_s_at	CDC42SE1	CDC42 small effector 1
594	0.0007993	-1.38	223225_s_at	SEH1L	SEH1-like (S. cerevisiae)
595	0.0007951	-1.4	231139_at	NA	NA
596	0.0007921	-3.08	206911_at	TRIM25	tripartite motif-containing 25
597	0.0007824	-1.55	1554390_s_at	ACTR2	ARP2 actin-related protein 2 homolog (yeast)
598	0.0007777	-1.32	208824_x_at	PCTK1	PCTAIRE protein kinase 1
599	0.0007732	-1.36	214258_x_at	KAT5	K(lysine) acetyltransferase 5
600	0.0007729	-1.47	208751_at	NAPA	N-ethylmaleimide-sensitive factor attachment protein, alpha
601	0.0007690	-1.16	238337_s_at	DNAJC21	DnaJ (Hsp40) homolog, subfamily C, member 21
602	0.0007673	-1.31	211314_at	CACNA1G	calcium channel, voltage-dependent, T type, alpha 1G subunit
603	0.0007657	-1.53	217834_s_at	SYNCRIP	synaptotagmin binding, cytoplasmic RNA interacting protein
604	0.0007625	-1.85	208653_s_at	CD164	CD164 molecule, sialomucin
605	0.0007608	-1.45	209091_s_at	SH3GLB1	SH3-domain GRB2-like endophilin B1
606	0.0007426	-1.25	1557533_at	NA	NA
607	0.0007405	-2.01	1555785_a_at	XRN1	5@#$%&-3@#$%& exoribonuclease 1
608	0.0007371	-1.41	236961_at	NA	NA
609	0.0007346	-1.14	204080_at	TOE1	target of EGR1, member 1 (nuclear)
610	0.0007341	-1.29	243852_at	LUC7L2	LUC7-like 2 (S. cerevisiae)
611	0.0007227	-1.29	210317_s_at	YWHAE	tyrosine 3-monooxygenase/tryptophan 5-monooxygenase activation protein, epsilon polypeptide
612	0.0007183	-1.4	209284_s_at	C3orf63	chromosome 3 open reading frame 63
613	0.0007169	-3.19	227361_at	HS3ST3B1	heparan sulfate (glucosamine) 3-O-sulfotransferase 3B1
614	0.0007139	-1.19	211066_x_at	PCDHGC3	protocadherin gamma subfamily C, 3
615	0.0006984	-1.09	202814_s_at	HEXIM1	hexamethylene bis-acetamide inducible 1
616	0.0006943	-1.35	226710_at	C8orf82	chromosome 8 open reading frame 82
617	0.0006932	-3.2	209969_s_at	STAT1	signal transducer and activator of transcription 1, 91kDa
618	0.0006882	-1.56	225242_s_at	CCDC80	coiled-coil domain containing 80
619	0.0006875	-1.5	214121_x_at	PDLIM7	PDZ and LIM domain 7 (enigma)
620	0.0006868	-1.41	203916_at	NDST2	N-deacetylase/N-sulfotransferase (heparan glucosaminyl) 2
621	0.0006826	-1.32	208901_s_at	TOP1	topoisomerase (DNA) I
622	0.0006769	-2.95	206028_s_at	MERTK	c-mer proto-oncogene tyrosine kinase
623	0.0006749	-1.35	205724_at	PKP1	plakophilin 1 (ectodermal dysplasia/skin fragility syndrome)
624	0.0006737	-1.23	228121_at	NA	NA
625	0.0006701	-1.04	226928_x_at	SLC25A37	solute carrier family 25, member 37
626	0.0006700	-1.37	1555301_a_at	DIP2A	DIP2 disco-interacting protein 2 homolog A (Drosophila)
627	0.0006616	-1.27	1566301_at	PPP1R11	protein phosphatase 1, regulatory (inhibitor) subunit 11
628	0.0006570	-1.46	234519_at	NOBOX	NOBOX oogenesis homeobox
629	0.0006553	-1.24	218520_at	TBK1	TANK-binding kinase 1
630	0.0006552	-1.55	201878_at	ARIH1	ariadne homolog, ubiquitin-conjugating enzyme E2 binding protein, 1 (Drosophila)
631	0.0006495	-1.3	1564131_a_at	NA	NA
632	0.0006472	-1.49	209102_s_at	HBP1	HMG-box transcription factor 1
633	0.0006450	-1.18	238586_at	LOC731489	hypothetical protein LOC731489
634	0.0006425	-1.03	216231_s_at	B2M	beta-2-microglobulin
635	0.0006398	-1.79	1552772_at	CLEC4D	C-type lectin domain family 4, member D
636	0.0006384	-1.42	201586_s_at	SFPQ	splicing factor proline/glutamine-rich (polypyrimidine tract binding protein associated)
637	0.0006373	-1.74	41644_at	SASH1	SAM and SH3 domain containing 1
638	0.0006346	-1.11	216652_s_at	DR1	down-regulator of transcription 1, TBP-binding (negative cofactor 2)
639	0.0006313	-1.3	212436_at	TRIM33	tripartite motif-containing 33
640	0.0006284	-1.57	212264_s_at	WAPAL	wings apart-like homolog (Drosophila)
641	0.0006259	-1.2	226481_at	VPRBP	Vpr (HIV-1) binding protein
642	0.0006104	-1.35	217490_at	NA	NA
643	0.0006091	-1.29	1557463_at	NA	NA
644	0.0005929	-1.35	238273_at	PL-5283	PL-5283 protein
645	0.0005927	-1.77	203840_at	BLZF1	basic leucine zipper nuclear factor 1
646	0.0005896	-1.17	237604_at	LOC415056	hypothetical gene LOC415056
647	0.0005883	-1.9	222881_at	HPSE	heparanase
648	0.0005855	-1.25	220634_at	TBX4	T-box 4
649	0.0005853	-1.3	200669_s_at	UBE2D3	ubiquitin-conjugating enzyme E2D 3 (UBC4/5 homolog, yeast)
650	0.0005801	-1.05	232017_at	TJP2	tight junction protein 2 (zona occludens 2)
651	0.0005772	-1.35	213918_s_at	NIPBL	Nipped-B homolog (Drosophila)
652	0.0005770	-1.62	215357_s_at	POLDIP3	polymerase (DNA-directed), delta interacting protein 3
653	0.0005750	-1.4	1561354_at	NA	NA
654	0.0005678	-1.08	206516_at	AMH	anti-Mullerian hormone
655	0.0005652	-2.05	211030_s_at	SLC6A6	solute carrier family 6 (neurotransmitter transporter, taurine), member 6
656	0.0005634	-2.18	222651_s_at	TRPS1	trichorhinophalangeal syndrome I
657	0.0005618	-2.05	57703_at	SENP5	SUMO1/sentrin specific peptidase 5
658	0.0005604	-11.89	211372_s_at	IL1R2	interleukin 1 receptor, type II
659	0.0005547	-1.2	1567246_at	OR5H1	olfactory receptor, family 5, subfamily H, member 1
660	0.0005488	-1.85	205003_at	DOCK4	dedicator of cytokinesis 4
661	0.0005371	-2.37	222262_s_at	ETNK1	ethanolamine kinase 1
662	0.0005358	-1.38	201684_s_at	TOX4	TOX high mobility group box family member 4
663	0.0005357	-1.85	206011_at	CASP1	caspase 1, apoptosis-related cysteine peptidase (interleukin 1, beta, convertase)
664	0.0005349	-1.16	211505_s_at	STAU1	staufen, RNA binding protein, homolog 1 (Drosophila)
665	0.0005342	-1.47	1554049_s_at	WDR42A	WD repeat domain 42A
666	0.0005321	-1.36	225978_at	FAM80B	family with sequence similarity 80, member B
667	0.0005228	-1.15	215056_at	NA	NA
668	0.0005162	-1.38	202066_at	PPFIA1	protein tyrosine phosphatase, receptor type, f polypeptide (PTPRF), interacting protein (liprin), alpha 1
669	0.0005087	-1.13	226128_at	NA	NA
670	0.0005005	-1.17	1562449_s_at	NA	NA
671	0.0004997	-1.51	217847_s_at	THRAP3	thyroid hormone receptor associated protein 3
672	0.0004992	-1.6	229845_at	MAPKAP1	mitogen-activated protein kinase associated protein 1
673	0.0004955	-2.82	211806_s_at	KCNJ15	potassium inwardly-rectifying channel, subfamily J, member 15
674	0.0004947	-2.32	217503_at	NA	NA
675	0.0004946	-1.2	221147_x_at	WWOX	WW domain containing oxidoreductase
676	0.0004897	-1.26	1552684_a_at	SENP8	SUMO/sentrin specific peptidase family member 8
677	0.0004707	-1.17	206251_s_at	AVPR1A	arginine vasopressin receptor 1A
678	0.0004609	-1.24	223546_x_at	LUC7L	LUC7-like (S. cerevisiae)
679	0.0004595	-1.33	208576_s_at	HIST1H3B	histone cluster 1, H3b
680	0.0004582	-1.97	202684_s_at	RNMT	RNA (guanine-7-) methyltransferase
681	0.0004567	-1.36	201378_s_at	UBAP2L	ubiquitin associated protein 2-like
682	0.0004553	-1.15	202178_at	PRKCZ	protein kinase C, zeta
683	0.0004545	-1.13	1555139_a_at	OTUD7B	OTU domain containing 7B
684	0.0004543	-1.47	244595_at	NA	NA
685	0.0004511	-1.32	210592_s_at	04/01/12	spermidine/spermine N1-acetyltransferase 1
686	0.0004444	-1.21	1554327_a_at	CANT1	calcium activated nucleotidase 1
687	0.0004435	-1.41	223430_at	SNF1LK2	SNF1-like kinase 2
688	0.0004430	-1.42	232437_at	CPSF3L	cleavage and polyadenylation specific factor 3-like
689	0.0004418	-1.11	202230_s_at	CHERP	calcium homeostasis endoplasmic reticulum protein
690	0.0004358	-1.8	211782_at	IDS	iduronate 2-sulfatase
691	0.0004288	-1.63	208869_s_at	GABARAPL1	GABA(A) receptor-associated protein like 1
692	0.0004258	-1.14	1554646_at	OSBPL1A	oxysterol binding protein-like 1A
693	0.0004189	-1.29	224410_s_at	LMBR1	limb region 1 homolog (mouse)
694	0.0004130	-1.09	201698_s_at	SFRS9	splicing factor, arginine/serine-rich 9
695	0.0004109	-1.57	218578_at	CDC73	cell division cycle 73, Paf1/RNA polymerase II complex component, homolog (S. cerevisiae)
696	0.0003904	-1.3	1566456_at	NA	NA
697	0.0003884	-2.78	226026_at	DIRC2	disrupted in renal carcinoma 2
698	0.0003884	-1.49	222035_s_at	PAPOLA	poly(A) polymerase alpha
699	0.0003882	-1.25	240313_at	DMRTB1	DMRT-like family B with proline-rich C-terminal, 1
700	0.0003880	-1.65	242943_at	ST8SIA4	ST8 alpha-N-acetyl-neuraminide alpha-2,8-sialyltransferase 4
701	0.0003868	-2.87	243271_at	NA	NA
702	0.0003850	-1.43	234173_s_at	NXF2	nuclear RNA export factor 2
703	0.0003839	-1.93	211368_s_at	CASP1	caspase 1, apoptosis-related cysteine peptidase (interleukin 1, beta, convertase)
704	0.0003823	-1.24	227712_at	LYRM2	LYR motif containing 2
705	0.0003803	-1.63	201377_at	UBAP2L	ubiquitin associated protein 2-like
706	0.0003785	-1.4	204858_s_at	TYMP	thymidine phosphorylase
707	0.0003695	-1.53	205415_s_at	ATXN3	ataxin 3
708	0.0003684	-2.46	209237_s_at	SLC23A2	solute carrier family 23 (nucleobase transporters), member 2
709	0.0003661	-1.25	235833_at	PPAT	phosphoribosyl pyrophosphate amidotransferase
710	0.0003575	-1.29	209590_at	BMP7	bone morphogenetic protein 7
711	0.0003529	-3.5	215966_x_at	GK3P	glycerol kinase 3 pseudogene
712	0.0003527	-1.22	202807_s_at	TOM1	target of myb1 (chicken)
713	0.0003455	-1.18	201198_s_at	PSMD1	proteasome (prosome, macropain) 26S subunit, non-ATPase, 1
714	0.0003409	-3.97	202068_s_at	LDLR	low density lipoprotein receptor
715	0.0003389	-1.07	200914_x_at	KTN1	kinectin 1 (kinesin receptor)
716	0.0003355	-1.77	225395_s_at	FAM120AOS	family with sequence similarity 120A opposite strand
717	0.0003300	-1.17	216306_x_at	PTBP1	polypyrimidine tract binding protein 1
718	0.0003300	-1.38	208985_s_at	EIF3J	eukaryotic translation initiation factor 3, subunit J
719	0.0003231	-1.15	1569932_at	NHSL2	NHS-like 2
720	0.0003229	-1.41	204781_s_at	FAS	Fas (TNF receptor superfamily, member 6)
721	0.0003210	-2	209593_s_at	TOR1B	torsin family 1, member B (torsin B)
722	0.0003206	-1.3	237285_at	SORBS2	sorbin and SH3 domain containing 2
723	0.0003169	-1.19	202550_s_at	VAPB	VAMP (vesicle-associated membrane protein)-associated protein B and C
724	0.0003084	-1.34	201461_s_at	MAPKAPK2	mitogen-activated protein kinase-activated protein kinase 2
725	0.0003068	-1.11	1563069_at	NA	NA
726	0.0002955	-1.14	218382_s_at	U2AF2	U2 small nuclear RNA auxiliary factor 2
727	0.0002940	-1.11	205570_at	PIP4K2A	phosphatidylinositol-5-phosphate 4-kinase, type II, alpha
728	0.0002921	-1.28	208642_s_at	XRCC5	X-ray repair complementing defective repair in Chinese hamster cells 5 (double-strand-break rejoining)
729	0.0002910	-1.24	221603_at	PEX16	peroxisomal biogenesis factor 16
730	0.0002863	-1.21	231211_s_at	LOC541469	hypothetical protein LOC541469
731	0.0002857	-2.02	219062_s_at	ZCCHC2	zinc finger, CCHC domain containing 2
732	0.0002808	-1.2	218570_at	KBTBD4	kelch repeat and BTB (POZ) domain containing 4
733	0.0002799	-1.16	223545_at	FANCD2	Fanconi anemia, complementation group D2
734	0.0002764	-1.34	1555614_at	SUGT1P	suppressor of G2 allele of SKP1 pseudogene (S. cerevisiae)
735	0.0002736	-1.53	1556283_s_at	FGFR1OP2	FGFR1 oncogene partner 2
736	0.0002703	-1.66	226022_at	SASH1	SAM and SH3 domain containing 1
737	0.0002699	-1.87	214838_at	SFT2D2	SFT2 domain containing 2
738	0.0002674	-1.26	206307_s_at	FOXD1	forkhead box D1
739	0.0002668	-1.41	208108_s_at	AVPR2	arginine vasopressin receptor 2
740	0.0002633	-1.21	239949_at	THNSL2	threonine synthase-like 2 (S. cerevisiae)
741	0.0002619	-1.76	1554096_a_at	RBM33	RNA binding motif protein 33
742	0.0002577	-1.25	203039_s_at	NDUFS1	NADH dehydrogenase (ubiquinone) Fe-S protein 1, 75kDa (NADH-coenzyme Q reductase)
743	0.0002569	-3.7	1563541_at	NA	NA
744	0.0002547	-1.33	210569_s_at	SIGLEC9	sialic acid binding Ig-like lectin 9
745	0.0002484	-1.44	203922_s_at	CYBB	cytochrome b-245, beta polypeptide
746	0.0002482	-1.33	220012_at	ERO1LB	ERO1-like beta (S. cerevisiae)
747	0.0002373	-2.41	236106_at	NA	NA
748	0.0002362	-1.34	242834_at	NA	NA
749	0.0002316	-1.2	220498_at	ACTL7B	actin-like 7B
750	0.0002303	-1.44	240873_x_at	DAB2	disabled homolog 2, mitogen-responsive phosphoprotein (Drosophila)
751	0.0002296	-1.19	221471_at	SERINC3	serine incorporator 3
752	0.0002267	-1.74	213236_at	SASH1	SAM and SH3 domain containing 1
753	0.0002262	-1.62	213988_s_at	04/01/12	spermidine/spermine N1-acetyltransferase 1
754	0.0002220	-1.2	239372_at	NA	NA
755	0.0002208	-1.35	240079_at	ZNF81	zinc finger protein 81
756	0.0002182	-1.65	205227_at	IL1RAP	interleukin 1 receptor accessory protein
757	0.0002149	-1.5	223751_x_at	TLR10	toll-like receptor 10
758	0.0002132	-1.35	1553677_a_at	TIPRL	TIP41, TOR signaling pathway regulator-like (S. cerevisiae)
759	0.0002103	-1.44	AFFX-HUMISGF3A/M97935_5_at	STAT1	signal transducer and activator of transcription 1, 91kDa
760	0.0002103	-1.11	202240_at	PLK1	polo-like kinase 1 (Drosophila)
761	0.0002099	-1.28	1556281_at	NA	NA
762	0.0002093	-1.53	222989_s_at	UBQLN1	ubiquilin 1
763	0.0002070	-1.16	204682_at	LTBP2	latent transforming growth factor beta binding protein 2
764	0.0002018	-1.37	225830_at	PDZD8	PDZ domain containing 8
765	0.0002016	-1.34	229208_at	CEP27	centrosomal protein 27kDa
766	0.0002006	-1.38	202211_at	ARFGAP3	ADP-ribosylation factor GTPase activating protein 3
767	0.0001969	-1.21	208696_at	CCT5	chaperonin containing TCP1, subunit 5 (epsilon)
768	0.0001905	-1.71	219207_at	EDC3	enhancer of mRNA decapping 3 homolog (S. cerevisiae)
769	0.0001804	-1.31	221248_s_at	WHSC1L1	Wolf-Hirschhorn syndrome candidate 1-like 1
770	0.0001758	-1.59	211781_x_at	NA	NA
771	0.0001730	-1.36	226037_s_at	TAF9B	TAF9B RNA polymerase II, TATA box binding protein (TBP)-associated factor, 31kDa
772	0.0001724	-1.14	200901_s_at	M6PR	mannose-6-phosphate receptor (cation dependent)
773	0.0001713	-1.67	213173_at	PCNX	pecanex homolog (Drosophila)
774	0.0001667	-2.06	206038_s_at	NR2C2	nuclear receptor subfamily 2, group C, member 2
775	0.0001616	-1.24	225397_at	C15orf57	chromosome 15 open reading frame 57
776	0.0001297	-2.14	211367_s_at	CASP1	caspase 1, apoptosis-related cysteine peptidase (interleukin 1, beta, convertase)
777	0.0001209	-1.66	222810_s_at	RASAL2	RAS protein activator like 2
778	0.0001196	-1.23	231718_at	SLU7	SLU7 splicing factor homolog (S. cerevisiae)
779	0.0001187	-1.32	223905_at	CCDC135	coiled-coil domain containing 135
780	0.0001127	-1.28	211672_s_at	ARPC4	actin related protein 2/3 complex, subunit 4, 20kDa
781	0.0001127	-1.38	200828_s_at	ZNF207	zinc finger protein 207
782	0.0001073	-1.29	244211_at	NA	NA
783	0.0001070	-12.04	205403_at	IL1R2	interleukin 1 receptor, type II
784	0.0001065	-1.83	209970_x_at	CASP1	caspase 1, apoptosis-related cysteine peptidase (interleukin 1, beta, convertase)
785	0.0001034	-6.35	217502_at	IFIT2	interferon-induced protein with tetratricopeptide repeats 2
786	0.0001029	-2.07	237867_s_at	PID1	phosphotyrosine interaction domain containing 1
787	0.0001028	-1.26	218516_s_at	IMPAD1	inositol monophosphatase domain containing 1
788	9.94E-005	-1.64	226312_at	RICTOR	rapamycin-insensitive companion of mTOR
789	9.41E-005	-1.2	210940_s_at	GRM1	glutamate receptor, metabotropic 1
790	9.02E-005	-1.36	1554556_a_at	ATP11B	ATPase, class VI, type 11B
791	8.89E-005	-1.14	226735_at	TAPT1	transmembrane anterior posterior transformation 1
792	8.70E-005	-3.84	213006_at	CEBPD	CCAAT/enhancer binding protein (C/EBP), delta
793	8.35E-005	-1.31	207787_at	KRT33B	keratin 33B
794	8.34E-005	-1.29	207410_s_at	TLX2	T-cell leukemia homeobox 2
795	8.21E-005	-1.6	223596_at	SLC12A6	solute carrier family 12 (potassium/chloride transporters), member 6
796	7.98E-005	-1.23	231859_at	C14orf132	chromosome 14 open reading frame 132
797	7.81E-005	-1.25	228277_at	FBXL19	F-box and leucine-rich repeat protein 19
798	7.75E-005	-1.35	210470_x_at	NONO	non-POU domain containing, octamer-binding
799	7.52E-005	-1.29	222432_s_at	CCDC47	coiled-coil domain containing 47
800	7.19E-005	-1.8	238496_at	NA	NA
801	7.11E-005	-1.38	208698_s_at	NONO	non-POU domain containing, octamer-binding
802	7.08E-005	-4.66	203946_s_at	ARG2	arginase, type II
803	7.03E-005	-1.17	1559952_x_at	LOC100132923	similar to hCG1993470
804	6.19E-005	-2.35	220104_at	ZC3HAV1	zinc finger CCCH-type, antiviral 1
805	5.57E-005	-2.43	203595_s_at	IFIT5	interferon-induced protein with tetratricopeptide repeats 5
806	5.53E-005	-1.33	1569859_at	NA	NA
807	5.31E-005	-1.5	224359_s_at	HOOK3	hook homolog 3 (Drosophila)
808	4.19E-005	-2.51	205921_s_at	SLC6A6	solute carrier family 6 (neurotransmitter transporter, taurine), member 6
809	3.18E-005	-2.18	205749_at	CYP1A1	cytochrome P450, family 1, subfamily A, polypeptide 1
810	2.93E-005	-1.38	1566136_at	NA	NA
811	2.02E-005	-1.51	210992_x_at	FCGR2C	Fc fragment of IgG, low affinity IIc, receptor for (CD32)
812	1.61E-005	-1.22	207801_s_at	RNF10	ring finger protein 10
813	1.32E-005	-2.03	222816_s_at	ZCCHC2	zinc finger, CCHC domain containing 2
814	1.29E-005	-1.25	211884_s_at	CIITA	class II, major histocompatibility complex, transactivator
815	8.60E-006	-1.68	212664_at	TUBB4	tubulin, beta 4
816	4.60E-006	-1.16	212081_x_at	BAT2	HLA-B associated transcript 2
817	3.60E-006	-1.21	1554177_a_at	ATP5S	ATP synthase, H+ transporting, mitochondrial F0 complex, subunit s (factor B)
818	7.00E-007	-1.82	224783_at	FAM100B	family with sequence similarity 100, member B
819	6.00E-007	-1.57	208840_s_at	G3BP2	GTPase activating protein (SH3 domain) binding protein 2
820	4.00E-007	-1.59	206717_at	MYH8	myosin, heavy chain 8, skeletal muscle, perinatal

Using the Gene Set Expression Comparison Analysis, as implemented in BRB Array tools, the Biocarta pathways that were associated with the differentially regulated genes were identified. Over 60 differentially regulated pathways were discovered in monocytes in response to LT treatment. As expected, the most significant pathway affected by LT treatment was the MAPK signaling pathway, with the p38 MAPK signaling pathway being most impacted with 103 genes affected (Figure 3B). Additional pathways altered by LT at the p < 0.001 significance level included the IL-18, Toll-Like Receptor, IFN alpha, and G-Protein Family signaling pathways. It is interesting to note that a previous study measuring the transcriptional response of human alveolar macrophages to anthrax spores detected an activation of the TLR pathways [22], and our results indicated anthrax LT targets 87 genes within the TLR signaling pathway (Figure 3B).

RGS14 is a protein involved in the regulation of G-protein signaling through attenuation of G-protein heterotrimer signaling, thereby inactivating this signaling cascade. The Affymetrix microarrays revealed that RGS14 expression in LT treated monocytes showed a 6 fold increase in expression (Table 2). This is a potentially significant finding in that RGS14 inhibits G-proteins important for chemotaxis. Therefore LT could be impairing chemotaxis not only by blocking Hsp27 phosphorylation through disruption of the p38 pathway [23], but also by causing over-expression of RGS14, thereby inhibiting G-protein mediated signaling required for actin-based motility.

**Table 2:** Predicted effects of LT on monocyte function.1

**Gene**	**Microarray**	**Effects**
RGS-14	5.61	Blockade of monocyte maturation to dendritic cells, inhibition of chemotaxis
CXCR2	5.04	Increased monocyte transendothelial migration into tissues
HPSE	-2.58	Diminished inflammatory response
CCR5	-2.33	Reduced responsiveness to the inflammatory mediators RANTES, MIP1 beta
ILIR2	-12.5	Increased IL1 alpha responsiveness and increased fever

1 Calculated fold changes compared to mock treated samples.

RGS14 expression is down-regulated during the maturation of monocytes to dendritic cells [24] and over-expression of this G-protein regulator would be expected to block monocyte maturation. RGS14 levels are also known to decrease in dendritic cells exposed to *Leishmania major* or *Toxoplasma gondii*, suggesting that RGS14 downregulation may be an important step in a normal immune response, and up-regulation of RGS14 by LT could be contributing to LT’s immunosuppressive effects [25].

Three chemokine receptors were also altered after LT treatment, suggesting that LT may be inducing functional defects in monocyte response signaling. IL-8 receptor beta (CXCR2) was up-regulated after LT treatment (Table 2). CXCR2 transduces signaling through a G-protein activated second messenger system. This receptor is important for monocyte transendothelial migration, and up-regulation of CXCR2 could serve to enhance the delivery of monocytes to tissues. Anthrax spores must be phagocytosed by macrophages in order to germinate into viable bacteria. An increase in the macrophage pool may aid in a reservoir for increased germination of viable bacteria. IL-1 receptor type II (IL-1R2), was found to be markedly down-regulated. IL-1R2 is a decoy receptor for IL-1 that functions either at the cell surface or in a soluble form [26]. The decreased expression of the decoy receptor would presumably increase IL-1a levels and increase the febrile response of the host potentially at least in part explaining the high fever that commonly accompanies systemic anthrax [27]. CCR5 is a receptor for the monocyte chemokines RANTES and MIP. The down-regulation of CCR5 by LT could reflect an inability of toxin-treated monocytes to differentiate into macrophages [28] (Table 2). During the early stages of infection, macrophages play a critical role in assisting *B. anthracis* pathogenesis by providing a place for bacteria germination from their spore form to viable bacteria. An increase in monocyte trafficking to allow an increase in spore uptake and subsequent germination would prove beneficial for *B. anthracis*. During later stages of infection, after release of viable bacteria, limiting monocyte differentiation to macrophages would assist in preventing clearance of viable bacteria.

In addition to an alteration in the chemokine response by LT, an additional enzyme, heparanase (HPSE), was found to be decreased in LT-treated human monocytes. This enzyme is an endoglycosidase that degrades heparin sulfate, resulting in disassembly of extracellular barriers required for cell migration [29]. Heparanase has also been postulated to play a role in inflammation [30] and our results showed a 2.6 fold decrease in heparanase gene expression (Table 2). One study has concluded that an *in vivo* siRNA against heparanase, along with an inhibitor of its enzymatic activity, results in a diminished inflammatory response [31]. Thus LT- mediated inhibition of heparanase expression could also contribute to the inhibition of the host immune response during an anthrax infection.

An external verification method using quantitative real-time PCR was utilized to confirm the microarray data. The eight genes corresponding to RGS14, IL8RB, TLR5, PPM1H, CD47, SYK, CCR5, and IL1R2 were chosen for microarray confirmation in monocytes. CCR5 and IL1R2 were confirmed to be down-regulated at 4 h after LT treatment, reinforcing the microarray data, while the other six genes were up-regulated, again confirming the microarray data (Table 3). A correlation curve was plotted (Figure 3C) and analyzed, showing a linear relationship between the microarray results and RT-PCR with a correlation coefficient of 0.885. Results were performed in duplicates and fold values were normalized to GAPDH. To exclude the possibility the lymphocyte contamination might be contributing to our microarray findings, a higher purity monocyte population (98% purity), obtained by adherence followed by washing off non-adherent lymphocytes, was treated with 500 ng/mL LT for 4 h and gene expression was assessed using real-time PCR. These experiments verified 3 genes to be increased after LT treatment: RGS14, TLR5, and CD47 (1.21-1.70), as observed by the microarray of suspended cells. These findings suggest that the changes in messenger RNA observed are primarily contributed by monocytes, but we cannot entirely exclude a contribution by lymphocytes.

**Table 3:** q-RTPCR confirmation of LT-induced genes.1

**Probe**	**Microarray**	**q-RT-PCR**	**Gene name**	**Primer sequence**
38290_at	5.61	7.40	RGS14-F	CAGGGATCTGTGAGAAACGAG
			RGS14-R	AGGTGATCCTGTTTTCCAGC
207008_at	5.04	7.50	IL8RB-F	GTCTAACAGCTCTGACTACCAC
			L8RB-R	TTAAATCCTGACTGGGTCGC
210166_at	3.90	2.24	TLR5-F	TTTTCAGGAGCCCGAGC
			TLR5-R	AGCCGAGATTGTGTCACTG
212686_at	2.65	3.85	PPM1H-F	GAGTACAGAGAAAGGAGCTTGG
			PPM1H-R	TCCAATAGTTGCCATTACCCG
226016_at	2.38	1.60	CD47-F	TTTGCTATACTCCTGTTCTGGG
			CD47-R	TGGGACGAAAAGAATGGCTC
209269_at	2.15	1.60	SYK-F	CAAGTTCTCCAGCAAAAGCG
			SYK-R	CATCCGCTCTCCTTTCTCTAAC
206991_at	-2.66	-2.33	CCR5-F	CCAAAAGCACATTGCCAAACG
			CCR5-R	ACTTGAGTCCGTGTCACAAGCC
205403_at	-12.5	-28.0	IL1R2-F	TGGCACCTACGTCTGCACTACT
			IL1R2-R	TTGCGGGTATGAGATGAACG

1 Calculated fold changes comparedtomock treated samples.

## Conclusions

Our investigations show human peripheral monocytes are susceptible to the actions of anthrax LT and do not undergo LT-mediated cytotoxicity after a four hour toxin treatment. We also find that LT induces changes in several genes involved in previously unidentified pathways including the TLR pathway, IFN alpha pathway, and G-Protein family signaling pathways. The identification of several previously unappreciated gene products including RGS14, IL8 receptor beta, CD47, TNF ligand, IL-16, Syk, CCR5, and IL-1 receptor II adds to our understanding of how LT impacts the immune response. Our pathway analysis reveals that anthrax LT targets multiple normal immune-regulatory pathways that would be expected to protect the host against anthrax infection. The increase in RGS14 levels and decrease in CCR5, along with IL-1R2, would likely impair monocyte functions and help to facilitate bacteria survival. *B. anthracis* maintains a selective advantage by impairing the host immune responses, thereby allowing for invasion and dissemination of the highly fatal bacilli. Our findings encourage further investigations into how these pathways converge functionally to impair normal monocyte function, along with providing new insights into the regulation of the host defense system and inflammation.

## Methods

### Monocyte isolation and toxin treatment

Whole blood was collected by venous puncture from healthy human volunteers into 8 mL vacutainer tubes containing Ficoll (BD Biosciences). The study followed US Department of Health and Human Services guidelines and was approved by the University of Florida Institutional Review Board. Whole blood was incubated with a monocyte negative selection antibody (Stem Cell Technologies) for 20 min., centrifuged 1700 × g for 25 min at RT, no brake over Ficoll, re-suspended in 10 mL RPMI (Mediatech) complete media, centrifuged at 250 × g for 9 min. to remove platelets, and re-suspended to 7-9 × 10^5^ cells/mL in RPMI. Monocytes were inverted at 37°C with 500 ng/mL LF and 500 ng/mL PA for 4 h. Additional qRT-PCR experiments were performed using higher monocyte purities (98%), obtained by first using a negative selection antibody cocktail (Stem Cell Technologies) isolation technique, followed by plastic adherence for 4 h, as described previously [32].

### Toxin purification

LF and PA were purified as previously described [33]. Briefly, *Bacillus anthracis* culture media was filtered through a 0.22 uM filter, followed by diethylaminoethyl cellulose (DEAE) anion exchange chromatography. The toxins were then subjected to gel filtration and hydrophobic interaction fast protein liquid chromatography (FPLC) and highly purified toxin components were confirmed by Coomassie Blue staining.

### Monocyte purity and apoptosis analysis

Monocytes were inverted at 37°C with 500 ng/mL LF and 500 ng/mL PA for 4 h, stained with CD-14 Pac Blue (BD Biosciences), Annexin-V-Fluorescein and propidium iodide (Roche). The cell population was gated first for CD14-Pac-Blue followed by analysis of the relative amount of Annexin (FL1) and PI (FL2) using flow cytometry FACScan (BD), and analyzed by FCS Express (De Novo).

### MEK cleavage

Purified monocytes were incubated at 37°C with 500 ng/mL lethal toxin for 4 h. Cells were lysed, ran on a 10% SDS-PAGE gel (Pierce), transferred to a PVDF membrane (Bio-rad) and probed for MEK1 (Upstate). Membranes were then stripped and probed for MEK3 (Santa Cruz). β-actin (Sigma) was used to check consistent loading amounts.

### RNA isolation

Purified monocytes from 4 healthy volunteers were incubated at 37°C with media alone or with 500 ng/mL LT for 4 h. Total RNA was collected using RNAeasy mini kit (Qiagen) and RNA quantity and quality was assessed using NanoDrop (Thermo Scientific) technology.

### Microarray procedure

100 ng total RNA was labeled using Affymetrix GeneChip® 3' IVT Express Kit for each replicate. Amplified labeled RNA was purified, fragmented, then hybridized for 16 h on Affymetrix GeneChips® (HG U133 plus 2.0) representing approximately 22,000 well-characterized human genes. Arrays were washed using Affymetrix GeneChip® Fluidics Station FS450 and scanned using GeneChip® Scanner 3000 7 G.

### Microarray analysis

Low-level analysis was performed using dChipmodeled-based expression matrix (dChip 2007 (DNA-Chip Analyzer), Build date: Jan 4, 2008). Unsupervised analysis - probes sets whose hybridization signal intensity exhibited a coefficient of variation of greater than 0.5 were analyzed by unsupervised hierarchical cluster analysis using algorithms implemented in dChip. Supervised analysis - significant probe sets between the treatment groups were identified using a paired *t*-test (by donor) at a significance threshold of p < 0.001. Leave-one-out-cross-validation using 4 prediction models was used to test the ability of probe sets significant at p < 0.001 to distinguish between the treatment groups. Microarray analyses were done using dCHIP and BRB-ArrayTools by Richard Simon ( http://linus.nci.nih.gov/BRB-ArrayTools.html). The microarray data for this study was deposited in the National Center for Biotechnology Information (NCBI) Gene Expression Omnibus (GEO) [30] with accession numbersGSM848717 through GSM 848724. The microarray data is also available in a series with accession number GSE34407.

### Quantitative real time-PCR (qRT-PCR)

RNA was collected using RNAeasy mini kit (Qiagen), quantitated using a Nanodrop system (Thermo Scientific), and 233 μg total RNA was used for cDNAsynthesis using SuperScript III First-Strand Synthesis (Invitrogen). cDNA was quantitated using SYBR Green JumpStart TaqReadyMix (Sigma) and 10 mM forward and 10 mM reverse primers were used for each indicated reaction. Primers used were as follows ACTB-F TCACCGAGCGCGGCT,ACTB-R TAATGTCACGCACGATTTCCC,GAPDH-F GGTGAAGGTCGGAGTCAACG, and GAPDH-R AGAGTTAAAAGCAGCCCTGGTG. All other primers are listed in Table 2. Reactions were run on the MJR Opticon Continuous Fluorescence detector (Bio-Rad) and analyzed with Opticon Monitor Software 1.08 (Bio-Rad).

## Authors’ contributions

KC performed the experiments, analyzed data, interpreted study, drafted, and wrote the manuscript. CL performed the gene expression profiling, biostatistical analysis, and helped design the study. SZ participated in the study design and assisted with experiments. GS participated in the design and interpretation of the study, as well as edited the manuscript. HB participated in the design and analyses of the study. CQ supplied the purified toxin and assisted in data interpretation. FS conceived the study and participated in the design and coordination, as well as edited the manuscript. All authors read and approved the final manuscript.
